# Malaria Surveillance — United States, 2015

**DOI:** 10.15585/mmwr.ss6707a1

**Published:** 2018-05-04

**Authors:** Kimberly E. Mace, Paul M. Arguin, Kathrine R. Tan

**Affiliations:** 1 Malaria Branch, Division of Parasitic Diseases and Malaria, Center for Global Health, CDC

## Abstract

**Problem/Condition:**

Malaria in humans is caused by intraerythrocytic protozoa of the genus *Plasmodium*. These parasites are transmitted by the bite of an infective female *Anopheles* species mosquito. The majority of malaria infections in the United States occur among persons who have traveled to regions with ongoing malaria transmission. However, malaria is occasionally acquired by persons who have not traveled out of the country through exposure to infected blood products, congenital transmission, laboratory exposure, or local mosquitoborne transmission. Malaria surveillance in the United States is conducted to provide information on its occurrence (e.g., temporal, geographic, and demographic), guide prevention and treatment recommendations for travelers and patients, and facilitate transmission control measures if locally acquired cases are identified.

**Period Covered:**

This report summarizes confirmed malaria cases in persons with onset of illness in 2015 and summarizes trends in previous years.

**Description of System:**

Malaria cases diagnosed by blood film microscopy, polymerase chain reaction, or rapid diagnostic tests are reported to local and state health departments by health care providers or laboratory staff members. Case investigations are conducted by local and state health departments, and reports are transmitted to CDC through the National Malaria Surveillance System (NMSS), the National Notifiable Diseases Surveillance System (NNDSS), or direct CDC consultations. CDC reference laboratories provide diagnostic assistance and conduct antimalarial drug resistance marker testing on blood samples submitted by health care providers or local or state health departments. This report summarizes data from the integration of all NMSS and NNDSS cases, CDC reference laboratory reports, and CDC clinical consultations.

**Results:**

CDC received reports of 1,517 confirmed malaria cases, including one congenital case, with an onset of symptoms in 2015 among persons who received their diagnoses in the United States. Although the number of malaria cases diagnosed in the United States has been increasing since the mid-1970s, the number of cases decreased by 208 from 2014 to 2015. Among the regions of acquisition (Africa, West Africa, Asia, Central America, the Caribbean, South America, Oceania, and the Middle East), the only region with significantly fewer imported cases in 2015 compared with 2014 was West Africa (781 versus 969). *Plasmodium falciparum*, *P. vivax*, *P. ovale,* and *P. malariae* were identified in 67.4%, 11.7%, 4.1%, and 3.1% of cases, respectively. Less than 1% of patients were infected by two species. The infecting species was unreported or undetermined in 12.9% of cases. CDC provided diagnostic assistance for 13.1% of patients with confirmed cases and tested 15.0% of *P. falciparum* specimens for antimalarial resistance markers. Of the U.S. resident patients who reported purpose of travel, 68.4% were visiting friends or relatives. A lower proportion of U.S. residents with malaria reported taking any chemoprophylaxis in 2015 (26.5%) compared with 2014 (32.5%), and adherence was poor in this group. Among the U.S residents for whom information on chemoprophylaxis use and travel region were known, 95.3% of patients with malaria did not adhere to or did not take a CDC-recommended chemoprophylaxis regimen. Among women with malaria, 32 were pregnant, and none had adhered to chemoprophylaxis. A total of 23 malaria cases occurred among U.S. military personnel in 2015. Three cases of malaria were imported from the approximately 3,000 military personnel deployed to an Ebola-affected country; two of these were not *P. falciparum* species, and one species was unspecified. Among all reported cases in 2015, 17.1% were classified as severe illnesses and 11 persons died, compared with an average of 6.1 deaths per year during 2000–2014. In 2015, CDC received 153 *P. falciparum*-positive samples for surveillance of antimalarial resistance markers (although certain loci were untestable for some samples); genetic polymorphisms associated with resistance to pyrimethamine were identified in 132 (86.3%), to sulfadoxine in 112 (73.7%), to chloroquine in 48 (31.4%), to mefloquine in six (4.3%), and to artemisinin in one (<1%), and no sample had resistance to atovaquone. Completion of data elements on the malaria case report form decreased from 2014 to 2015 and remains low, with 24.2% of case report forms missing at least one key element (species, travel history, and resident status).

**Interpretation:**

The decrease in malaria cases from 2014 to 2015 is associated with a decrease in imported cases from West Africa. This finding might be related to altered or curtailed travel to Ebola-affected countries in in this region. Despite progress in reducing malaria worldwide, the disease remains endemic in many regions, and the use of appropriate prevention measures by travelers is still inadequate.

**Public Health Actions:**

The best way to prevent malaria is to take chemoprophylaxis medication during travel to a country where malaria is endemic. As demonstrated by the U.S. military during the Ebola response, use of chemoprophylaxis and other protection measures is possible in stressful environments, and this can prevent malaria, especially *P. falciparum,* even in high transmission areas. Detailed recommendations for preventing malaria are available to the general public at the CDC website (https://www.cdc.gov/malaria/travelers/drugs.html). Malaria infections can be fatal if not diagnosed and treated promptly with antimalarial medications appropriate for the patient’s age and medical history, the likely country of malaria acquisition, and previous use of antimalarial chemoprophylaxis. Health care providers should consult the CDC *Guidelines for Treatment of Malaria in the United States* and contact the CDC’s Malaria Hotline for case management advice when needed. Malaria treatment recommendations are available online (https://www.cdc.gov/malaria/diagnosis_treatment) and from the Malaria Hotline (770-488-7788 or toll-free at 855-856-4713). Persons submitting malaria case reports (care providers, laboratories, and state and local public health officials) should provide complete information because incomplete reporting compromises case investigations and efforts to prevent infections and examine trends in malaria cases. Compliance with recommended malaria prevention strategies is low among U.S. travelers visiting friends and relatives. Evidence-based prevention strategies that effectively target travelers who are visiting friends and relatives need to be developed and implemented to reduce the numbers of imported malaria cases in the United States. Molecular surveillance of antimalarial drug resistance markers (https://www.cdc.gov/malaria/features/ars.html) has enabled CDC to track, guide treatment, and manage drug resistance in malaria parasites both domestically and internationally. More samples are needed to improve the completeness of antimalarial drug resistance marker analysis; therefore, CDC requests that blood specimens be submitted for all cases diagnosed in the United States.

## Introduction

Malaria parasites of the *Plasmodium* genus are transmitted through the bite of infected mosquitoes. Female *Anopheles* species mosquitoes transmit four *Plasmodium* species that commonly cause illness in humans: *P. falciparum*, *P. vivax, P. ovale*, and *P. malariae.* Mixed infections with multiple species are possible and occur in areas where more than one species is in circulation (*1*). Rarely, humans can be infected with *P. knowlesi,* a predominantly simian malaria found in Southeast Asia. In 2015, malaria was endemic in 91 countries and territories in the tropics and subtropics, with approximately half the world population at risk for infection. The World Health Organization (WHO) estimated that 212 million cases of malaria occurred worldwide in 2015, resulting in 429,000 deaths (*2*). The African region accounts for an estimated 92% of all malaria deaths (*2*). *P. falciparum* and *P. vivax* contribute the most morbidity worldwide. *P. falciparum* is the most pathogenic malaria species, has the highest prevalence in sub-Saharan Africa, and is most commonly associated with severe illness and death, typically among children aged <5 years. *P. vivax* is less prevalent in sub-Saharan Africa because much of the population lacks the Duffy antigen classically associated with *P. vivax* invasion of red blood cells. Because of its ability to survive at lower temperatures and higher elevations, *P. vivax* has a broader geographic range than *P. falciparum,* and outside of the African region, *P. vivax* is estimated to account for 41% of malaria infections (*2*). In some temperate regions, *P. vivax* infections can have incubation periods of ≥6 months between inoculation and first symptom onset (*3*–*5*). Malaria relapses are common with *P. vivax* and *P. ovale* parasites, which have dormant liver stages (hypnozoites) that can reactivate months or years after the acute infection. *P. malariae* parasites mature slowly in human and mosquito hosts, and although they do not typically cause severe symptoms, they can result in persistent low-density infections that can last for years or even a lifetime (*6*). 

Through the mid-20th century, malaria was endemic in much of the United States,* with approximately 300 cases per 100,000 population in 1920 (*7*). By 1942, malaria was limited to the southeastern United States, where the Office of Malaria Control in War Areas, the precursor to CDC, was established to reduce the impact of vectorborne diseases, especially malaria. Transmission of malaria was interrupted because of improved housing and socioeconomic conditions and focused efforts that started in the late 1940s including case management, vector control, and environmental management (*8*). Since 1957, malaria surveillance has been supported to detect cases and prevent reintroduction, monitor antimalarial resistance, assess trends in case acquisition, and guide malaria prevention and treatment recommendations for U.S. residents. Most malaria cases diagnosed in the United States are imported from countries with ongoing mosquitoborne transmission. Occasionally, congenital transmission occurs or induced cases result from exposure to blood products. As of 2015, the last case of transfusion-acquired malaria had occurred in 2011 (*9*). Malaria vectors exist throughout the United States (*10*). Consequently, state and local health departments and CDC investigate cryptic cases for which exposure cannot be explained. The most recent occurrence of local mosquitoborne transmission occurred in 2003, when eight cases were diagnosed in Palm Beach, Florida (*11*,*12*).

Clinical illness results from the presence of an asexual, intraerythrocytic stage of the parasite in red blood cells; symptom severity ranges from absent or mild symptoms to severe illness and death. Factors that contribute to variability in illness severity are complex and include the parasite species; the patient’s age, immune status, general health, and nutritional constitution; chemoprophylaxis effects; and time to initiate appropriate treatment (*6*). Malaria symptoms vary; however, the majority of patients have fever (*13*). Symptoms associated with uncomplicated malaria include chills, sweating, headache, fatigue, myalgia, cough, and nausea. If not treated promptly, malaria can affect multiple organ systems and result in altered consciousness (cerebral malaria), acute kidney injury and liver failure, respiratory distress, coma, permanent disability, and death. Travel history should be routinely requested for patients who have fever in the United States. Malaria should be considered in the differential diagnosis for all persons who have fever and who recently traveled to areas where malaria is endemic, as well as for persons who have fever of unknown origin, regardless of travel history. This report, which is intended for public health authorities, summarizes malaria cases reported to CDC with onset of symptoms in 2015, describes trends during previous years, and highlights information on risk factors and prevention. Information on chemoprophylaxis, diagnosis, and treatment is provided for health care professionals, as are additional resources for malaria information. 

## Methods

### Data Sources and Analysis

Malaria case reports were submitted to CDC through the National Malaria Surveillance System (NMSS) and the National Notifiable Diseases Surveillance System (NNDSS) (*14*). Although both systems rely on passive reporting, the number of cases might vary because of differences in date classifications. NNDSS report dates are assigned according to the date reported to the health department, and NMSS assigns dates according to illness onset. In addition, NNDSS provides only basic case demographic information, whereas NMSS collects detailed epidemiologic data, including laboratory confirmation, travel history, and clinical history, which facilitate investigation and classification of each case. Typically, NMSS cases are reported by health care providers or laboratories to local or state health departments and then to CDC. Some cases also are reported through direct consultation with CDC malaria staff via the Malaria Hotline or sent to CDC directly from clinics, health care providers, and laboratories. Diagnostic confirmation of cases often is facilitated by the CDC reference laboratory. The Armed Forces Health Surveillance Branch provides military malaria case reports to NMSS. This report summarizes data from the integration of all NMSS and NNDSS cases and CDC reference laboratory reports after deduplication and reconciliation.

Malaria cases are classified as confirmed or suspected using the 2014 case definition case definition from the Council of State and Territorial Epidemiologists and CDC (*15*). Malaria cases are further categorized by infecting species: *P. falciparum*, *P. vivax*, *P. malariae*, and *P. ovale*. When more than a single species is detected, the case is categorized as a mixed infection. All categories are mutually exclusive. Diagnosis of malaria is made by blood film microscopy or polymerase chain reaction (PCR). A rapid diagnostic test (RDT) can be used to detect malaria antigens (*16*); however, the diagnosis must be confirmed either by microscopy or PCR to be counted as a case. Only data from confirmed cases are included in this report. Five suspected cases were omitted from analysis because they were tested by RDT only and not validated with microscopy or PCR as required in the case definition for confirmed malaria. 

CDC staff members review all reports when received and request any additional information needed from the provider or the jurisdiction. Cases classified as being acquired in the United States are investigated further, in addition to those classified as induced, congenital, introduced, or cryptic according to the definitions that follow. Instructions for completing the malaria case report form, including the applicable definitions, are available from the CDC malaria website (*17*). Information from the structured malaria case report form is entered into a database (*18*). Data elements analyzed include age, sex, pregnancy status, residence, illness onset date, laboratory results (test type, species, and parasitemia percentage), travel history (countries and dates), chemoprophylaxis (medication used and adherence), history of malaria (date and species), blood transfusion or organ transplant history, clinical complications, treatment medications, deaths, and case classification.

The chi-square test was used to calculate p values and assess differences between variables reported in 2015 compared with previous years. A p value of <0.05 was considered statistically significant. Linear regression using least-squares methods (the Pearson product-moment correlation coefficient, R^2^) was used to assess the linear trend in the number of cases during 1973–2015 and the number of airline flights from the United States to Africa.

### Definitions

The following definitions are used in malaria surveillance for the United States (*15*,*17*).

- **U.S. residents:** Persons who live in the United States, including both civilian and U.S. military personnel, regardless of legal citizenship.
- **U.S. civilians:** Any U.S. residents, excluding U.S. military personnel.
- **Foreign residents:** Persons who are residents of a country other than the United States.
- **Travelers visiting friends or relatives (VFR):** Immigrants, ethnically and racially distinct from the major population of the country of residence (a country where malaria is not endemic), who return to their homeland (a country where malaria is endemic) to visit friends or relatives; includes family members (e.g., spouse or children) who were born in the country of residence (*19*).
- **Laboratory criteria for diagnosis:** Demonstration of malaria parasites on blood film, by PCR, or by RDT (followed by microscopy or PCR confirmation).
- **Confirmed case:** Symptomatic or asymptomatic infection that occurs in a person in the United States or one of its territories who has laboratory-confirmed (by microscopy or PCR) malaria parasitemia, regardless of whether the person had previous episodes of malaria while in other countries. A subsequent episode of malaria is counted as an additional case, regardless of the detected *Plasmodium* species, unless the case is indicated as a treatment failure.
- **Suspect case:** Symptomatic or asymptomatic infection that occurs in a person in the United States or one of its territories who has *Plasmodium* species infection detected by RDT without confirmation by microscopy or PCR, regardless of whether the person experienced previous episodes of malaria while in other countries.
- **Adherence to chemoprophylaxis:** Self-reported response to the question, “Was chemoprophylaxis taken as prescribed?”
- **Treatment according to CDC recommendations (i.e., appropriate treatment):** Treated with a CDC-recommended regimen appropriate for species, region, and severity of disease (*20*). Patients who received more antimalarial medication than recommended were classified as appropriately treated because the precise sequence and circumstances of excess treatment are not included in the malaria case report and characterizing the purpose or appropriateness of the additional antimalarial treatment is not possible.

This report also uses terminology derived from the recommendations of the World Health Organization (*21*). Definitions of the following terms are included for reference:

- **Indigenous malaria.** Local mosquitoborne transmission of malaria with no evidence of importation and no direct link to transmission from an imported case.
- **Introduced malaria.** Local mosquitoborne transmission of malaria with strong epidemiological evidence linking the case to an imported case.
- **Congenital malaria:** Malaria infection transmitted directly from mother to child during pregnancy or childbirth.
- **Imported malaria:** Malaria acquired outside a specific area. In this report, imported cases are those acquired outside the United States and its territories.
- **Induced malaria:** Malaria transmission through a blood transfusion, organ transplantation, or another parenteral route, not mosquitoborne or congenital transmission.
- **Relapsing malaria:** Recurrence of disease after it has been apparently cured. In malaria, true relapses are caused by reactivation of dormant liver-stage parasites (hypnozoites) of *P. vivax* and *P. ovale* only. Acute illness typically occurs within 45 days of exposure; therefore, likely relapses of *P. vivax* and *P. ovale* are defined as occurring >45 days after travel to an area where malaria is endemic.
- **Severe malaria:** A case of malaria with one or more of the following manifestations: neurologic symptoms, acute kidney injury, severe anemia (hemoglobin [Hb] <7g/dL), acute respiratory distress syndrome (ARDS), jaundice, or ≥5% parasitemia (*22*). Cases also were counted as severe if the person received treatment for severe malaria (i.e., artesunate, quinidine, or an exchange blood transfusion) despite having no specific severe manifestations reported. All fatal malaria cases were classified as severe.
- **Cryptic malaria:** A case of malaria for which epidemiologic investigations cannot identify a plausible mode of acquisition.

### Diagnosis of Malaria

To diagnose malaria promptly, health care providers must obtain a travel history from every patient who has fever. Malaria should be included in the differential diagnosis for every patient with fever who has traveled to an area where malaria is endemic and for patients with fever of unknown origin, regardless of travel history. Three laboratory tests are available to diagnose malaria, including peripheral blood smear, PCR, and RDT; however, blood smear is the gold standard. If malaria is suspected, a Giemsa-stained film of the patient’s peripheral blood should be examined by microscopy for parasites as soon as possible. Thick and thin blood films must be prepared correctly because diagnostic accuracy depends on blood film quality and examination by experienced laboratory personnel (*23*,*24*). This test can quickly detect the presence of malaria parasites and can be used to determine the species and percentage of red blood cells that are infected, which are all essential to guiding appropriate treatment of persons infected with malaria. Three sets of thick and thin blood films spaced 12–24 hours apart should be done before ruling out malaria. During the Ebola virus disease (Ebola) outbreak in West Africa that began in 2014, concern was expressed that the Ebola virus might not be inactivated by the smear preparation process. As a result, CDC developed additional steps to inactivate viruses, including Ebola, during the slide preparation process (*25*). PCR is useful to confirm the species and to guide treatment, especially to prevent relapses from *P. vivax* and *P. ovale* infections. In most laboratories where PCR is available, it cannot be performed quickly enough to be useful in the initial diagnosis and treatment of acute malaria. PCR should be performed for all cases of malaria diagnosed in the United States to confirm the infecting species (*15*,*26*).

The BinaxNOW malaria RDT (Inverness Medical Professional Diagnostics, Scarborough, Maine) detects circulating malaria-specific antigens and is approved for use by hospital and commercial laboratories. Therefore, the test should be used in a clinical laboratory by trained staff members and not by clinicians or the general public (*16*,*27*). In the United States, use of RDTs can decrease the amount of time required to determine whether a patient is infected with malaria but does not eliminate the need for standard blood film tests. RDTs are not able to determine all *Plasmodium* species or quantify malaria parasites. Positive RDT results must be confirmed by microscopy (*16*) to provide additional information about species and density of infection. If microscopy is not performed, then PCR can confirm an RDT result and determine the species. Because RDTs are less sensitive than microscopy, negative RDT results should also be confirmed by microscopy or PCR.

### Drug Resistance Marker Surveillance

In 2012, CDC began molecular surveillance for malaria drug resistance markers, with the goal of detecting and characterizing malaria parasites that carry genetic markers (typically single nucleotide polymorphisms [SNPs] in one or more loci) associated with drug resistance. The surveillance data will help identify where drug-resistant foci might be present or emerging in specific parts of the world where malaria is endemic. For each sample submitted, species confirmation testing is conducted using a duplex real-time PCR capable of detecting the four human-infecting *Plasmodium* species. Molecular genotyping for known markers of drug resistance (typically SNPs in known genes) are investigated for all *P. falciparum* samples. Additional species will be similarly evaluated as new laboratory methods are developed. Each sample submitted is tested for molecular markers associated with resistance to sulfadoxine, pyrimethamine, chloroquine, mefloquine, atovaquone, and artemisinin.

Samples for molecular resistance monitoring are processed for PCR amplification of parasite DNA using appropriate primers for the genes of interest and sequenced by the Sanger method using the ABI 3130 capillary sequencer (Thermo Fisher Scientific, Waltham, Massachusetts) according to described methods (*28*). Fragments of genes encoding molecular targets of chloroquine (chloroquine resistance transporter gene, *pfcrt*), pyrimethamine (dihydrofolate reductase gene, *dhfr*), sulfadoxine (dihydropteroate synthase gene, *dhps*), atovaquone (cytochrome b gene, *pfcytb*), mefloquine (multidrug resistance 1 protein gene, *pfmdr-1* and *pfmdr-1* copy number), and artemisinin (kelch K13-propeller domain) are analyzed for SNPs by comparing each sequence to the reference genome.

**Chloroquine resistance markers.** The *pfcrt* gene sequence is analyzed to identify polymorphism at codons C72S, M74I, N75E, and K76T.
**Pyrimethamine resistance markers.** The *pfdhfr* gene sequence is analyzed to identify polymorphism at codons A16V, C50R, N51I, C59R, S108T/N, and I164L.
**Sulfadoxine resistance markers.** The *pfdhps* gene sequence is analyzed to identify polymorphism at codons S436A, A437G, and K540E.
**Atovaquone resistance markers.** The *pfcytb* gene sequence is analyzed to identify polymorphism at codons I258M and Y268S (*29*).
**Mefloquine resistance markers.** A real-time PCR assay is used to determine the *pfmdr-1* copy number variation in the *P. falciparum* samples using the comparative cycle threshold (ΔΔC_T_) method as previously described (*30*). DNA from the 3D7 laboratory control, which has a single copy of *pfmdr-1,* was used as the calibrator. In addition, DNA from Indochina W2mef and Dd2 are used as multiple copy number controls.
**Artemisinin resistance markers:** The K13-propeller domain for artemisinin resistance is amplified using a nested PCR method previously described (*31*,*32*). The sequence data are analyzed using Geneious Pro R8 (Biomatters, Auckland, New Zealand) to identify polymorphisms associated with resistance.

## Results

### General Surveillance

**FIGURE 1:**
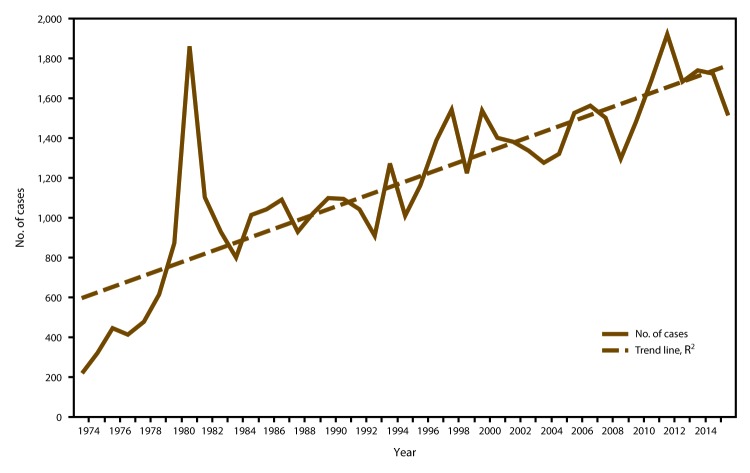
Number of malaria cases among U.S. and foreign residents — United States, 1973–2015* **Abbreviation: **R^2^ = square of the Pearson product moment correlation coefficient. * R^2^ = 0.695 is the average rate in increase of cases over time.

**FIGURE 2:**
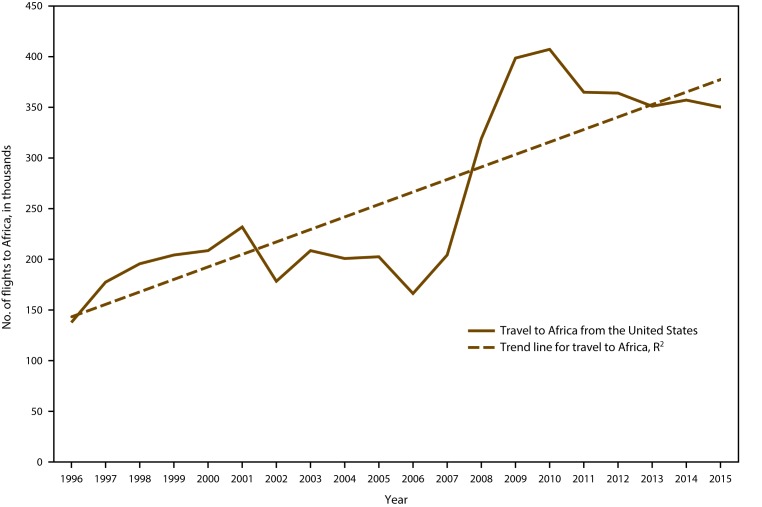
Number of airline flights from the United States to Africa* by U.S. citizens — 1996–2015^^†^^ **Abbreviation: **R^2^ = square of the Pearson product moment correlation coefficient. * Travel to Africa: R^2^ = 0.703. ^†^ **Source:** National Travel and Tourism Office. International air travel statistics. Washington, DC. https://travel.trade.gov/research/monthly/departures/index.asp

CDC received 1,517 reports of confirmed malaria cases among persons in the United States and its territories with onset of symptoms during 2015, which represents a 12.1% decrease in confirmed malaria cases compared with 2014 (n = 1,725).^†^ The highest number of malaria cases reported since 1985 occurred in 2011, with 1,925 cases, whereas an average of 1,718 annual cases were reported during 2012–2014. The decrease in cases in 2015 correlates with the decrease in cases imported from West Africa (see Region of Acquisition and Diagnosis) and could be related to altered travel because of the Ebola epidemic (*33*,*34*). Since 1973, the overall trend has been an increasing number of malaria cases, with an average gain of 27.8 cases per year (R^2^ = 0.695) (Figure 1). This increase in malaria cases coincides with the increasing trend in the annual number of international airline flights among U.S. citizens. According to data from the National Travel and Tourism Office, during 1996–2015, an average annual addition of approximately 521,000 trips occurred to all destinations (R^2^ = 0.742), and an average annual gain of approximately 12,000 to Africa (R^2^ = 0.703) (Figure 2) (*35*). In 2015, a total of 952 (62.8%) cases were among U.S. residents, 367 (24.2%) were among foreign residents, and 198 (13.1%) were among patients with unknown or unreported resident status (Table 1).

**TABLE 1:** Number of malaria cases* among U.S. military personnel, U.S. civilians, and foreign residents — United States, 1970–2015

Year	U.S. military personnel	U.S. civilians	Foreign residents	Status not recorded	Total
1970	4,096	90	44	17	**4,247**
1971	2,975	79	69	57	**3,180**
1972	454	106	54	0	**614**
1973	41	103	78	0	**222**
1974	21	158	144	0	**323**
1975	17	199	232	0	**448**
1976	5	178	227	5	**415**
1977	11	233	237	0	**481**
1978	31	270	315	0	**616**
1979	11	229	634	3	**877**
1980	26	303	1,534	1	**1,864**
1981	21	273	809	0	**1,103**
1982	8	348	574	0	**930**
1983	10	325	468	0	**803**
1984	24	360	632	0	**1,016**
1985	31	446	568	0	**1,045**
1986	35	410	646	0	**1,091**
1987	23	421	488	0	**932**
1988	33	550	440	0	**1,023**
1989	35	591	476	0	**1,102**
1990	36	558	504	0	**1,098**
1991	22	585	439	0	**1,046**
1992	29	394	481	6	**910**
1993	278	519	453	25	**1,275**
1994	38	524	370	82	**1,014**
1995	12	599	461	95	**1,167**
1996	32	618	636	106	**1,392**
1997	28	698	592	226	**1,544**
1998	22	636	361	208	**1,227**
1999	55	833	381	271	**1,540**
2000	46	827	354	175	**1,402**
2001	18	891	316	158	**1,383**
2002	33	849	272	183	**1,337**
2003	36	767	306	169	**1,278**
2004	32	775	282	235	**1,324**
2005	36	870	297	325	**1,528**
2006	50	736	217	561	**1,564**
2007	33	701	263	508	**1,505**
2008	19	510	176	593	**1,298**
2009	18	661	201	604	**1,484**
2010	46	1,085	368	192	**1,691**
2011	91	1,098	386	350	**1,925**
2012	43	1,121	328	195	**1,687**
2013	14	1,136	349	242	**1,741**
2014	31	1,114	384	196	**1,725**
2015	23	929	367	198	**1,517**

*A case was defined as symptomatic or asymptomatic illness that occurs in the United States or one of its territories in a person who has laboratory-confirmed malaria parasitemia (microscopy or PCR), regardless of whether the person had previous attacks of malaria while in other countries. A subsequent attack of malaria occurring in a person is counted as an additional case if the demonstrated *Plasmodium* species differs from the initially identified species or if it is indicated as a relapsing infection demonstrating the same *Plasmodium* species as identified previously. If a subsequent attack of malaria occurs as a result of a drug failure caused by a resistant parasite, then the case is not counted as an additional case.

Among the 1,517 confirmed cases, one was classified as congenital, 1,485 were imported from countries in which malaria is endemic, and 31 case reports had an incomplete travel history, which prevented classification. Of the 31 cases with unknown classification, 10 had laboratory reports only, which provided diagnostic test information and demographic data; 16 cases were reported through NNDSS with basic demographic data; and five were reported by local or state health departments as lost to follow-up with insufficient information to classify importation status. No case in 2015 was reported as having been acquired by local transmission in the United States.

### *Plasmodium* Species

In 2015, a total of 1,322 (87.2%) cases reported to CDC included information on the infecting *Plasmodium* species, which is similar to the proportion from 2014 (88.3%). Specimens from 198 (13.1%) cases were sent to the CDC reference laboratory for diagnostic assistance in 2015, and CDC identified the species for 195 (98.5%) specimens. Among these 195 specimens, CDC determined or corrected the species for 108 (55.4%) (Table 2). No *Plasmodium* species was detected in an additional 447 specimens submitted to CDC in 2015.

**TABLE 2:** Comparison of malaria species reported on the specimen submission form and CDC laboratory results* — United States, 2015

Species reported on the specimen submission form	Species identified by the CDC reference laboratory						Total
	*P. falciparum*	*P. vivax*	*P. ovale*	*P. malariae*	Mixed	*Plasmodium *unspecified	
*P. falciparum*	75	0	1	1	1	0	**78**
*P. vivax*	0	6	1	0	0	1	**8**
*P. ovale*	0	0	3	0	0	0	**3**
*P. malariae*	4	0	0	3	0	1	**8**
Mixed *Plasmodium* species	0	0	0	0	0	0	**0**
Unknown/Missing	60^†^	16	16	7	1	1	**101**
**Total**	**139**	**22**	**21**	**11**	**2**	**3**	**198**

*Of the 195 samples with a CDC-determined species, CDC corrected or provided the species information for 108 (55.4%) specimens.

^†^Includes one sample reported as a non-*Plasmodium* species on the specimen submission form.

Among the 1,322 cases with a species determination, the majority were *P. falciparum* (n = 1,023 [77.4%]); *P. vivax* infections were the second most common, with 13.5% (n = 178) of cases reported in 2015 (Table 3). The species distribution in 2015 for *P. falciparum*, *P. vivax*, *P. ovale*, *P. malariae*¸ and mixed *Plasmodium* species infections was similar to that in 2014. 

**TABLE 3:** Number of malaria cases, by *Plasmodium* species and year — United States, 2011–2015

*Plasmodium* species	2011	2012	2013	2014	2015
	No. (%)	No. (%)	No. (%)	No. (%)	No. (%)
*P. falciparum*	948 (49.3)	985 (58.4)	1,059 (60.8)	1,141 (66.1)	1,023 (67.4)
*P. vivax*	420 (21.8)	280 (16.6)	245 (14.1)	230 (13.3)	178 (11.7)
*P. ovale*	51 (2.6)	59 (3.5)	65 (3.7)	90 (5.2)	62 (4.1)
*P. malariae*	50 (2.6)	54 (3.2)	45 (2.6)	47 (2.8)	47 (3.1)
Mixed	21 (1.1)	21 (1.2)	41 (2.3)	15 (0.9)	12 (0.8)
Undetermined	435 (22.6)	288 (17.1)	286 (16.4)	202 (11.7)	195 (12.9)
**Total**	**1,925 (100)**	**1,687 (100)**	**1,741 (100)**	**1,725 (100)**	**1,517 (100)**

Information on the infecting species and region of acquisition was included for 1,264 case reports; of these 1,078 (85.3%) were from Africa. Information on region of acquisition information was available for 984 *falciparum* cases; 934 (94.9%) were acquired in Africa, which was significantly lower than in 2014 (1,074 of 1,099, 97.7%). *P. falciparum* infections accounted for 86.6% of cases from Africa, 4.8% from Asia, 70.9% from Central America and the Caribbean, and 20.8% from South America (Table 4). The number of *P. falciparum* cases imported from Central America and the Caribbean increased significantly from six in 2014 to 39 in 2015, which was related to the increase in the total number of cases imported from this region in 2015 (25 in 2014 and 66 in 2015). The increase in *P. falciparum* infections imported from this region resulted from cases acquired from the island of Hispaniola. The number of cases from Haiti decreased from a peak of 171 cases in 2010 to five in 2014; in 2015, the number of malaria cases from Haiti was 11. During 2005–2014, the United States had an average of 4.6 cases per year imported from the Dominican Republic. In 2014, two cases were imported from the Dominican Republic, and the number increased significantly to 32 cases in 2015.

**TABLE 4:** Number of imported malaria cases, by country of acquisition and *Plasmodium* species — United States, 2015

Country of acquisition	*P. falciparum*	*P. vivax*	*P. ovale*	*P. malariae*	Mixed	Unknown	Total
**Africa**	**934**	**39**	**56**	**39**	**10**	**132**	**1,210**
Angola	4	0	0	0	1	0	**5**
Benin	4	0	0	0	0	0	**4**
Burkina Faso	21	1	1	0	0	1	**24**
Burundi	1	0	0	0	0	0	**1**
Cameroon	69	2	3	0	1	9	**84**
Central African Republic	6	0	0	0	0	1	**7**
Chad	9	0	0	0	0	1	**10**
Congo, Republic Of	26	0	2	1	0	4	**33**
Côte d’Ivoire	74	1	4	2	0	6	**87**
Equatorial Guinea	1	0	0	0	0	0	**1**
Eritrea	0	2	0	1	1	1	**5**
Ethiopia	4	6	0	0	0	9	**19**
Gabon	1	0	0	0	0	0	**1**
Gambia	7	0	0	0	0	0	**7**
Ghana	125	2	3	5	0	12	**147**
Guinea	34	0	1	2	0	3	**40**
Guinea-Bissau	3	0	0	0	0	0	**3**
Kenya	43	2	4	4	0	6	**59**
Liberia	46	1	9	3	0	8	**67**
Malawi	12	0	0	1	0	1	**14**
Mali	13	0	0	0	0	0	**13**
Mauritania	0	1	0	0	0	0	**1**
Mozambique	7	1	0	2	1	1	**12**
Niger	3	0	0	0	0	1	**4**
Nigeria	224	8	15	3	3	37	**290**
Rwanda	5	0	1	1	0	2	**9**
Senegal	13	0	0	0	0	1	**14**
Sierra Leone	32	1	1	1	0	6	**41**
Somali Republic	1	0	0	0	0	0	**1**
South Africa	0	0	0	0	0	1	**1**
South Sudan	9	1	0	0	0	2	**12**
Sudan	24	6	2	1	1	1	**35**
Tanzania	18	0	1	3	0	1	**23**
Togo	18	1	3	1	0	4	**27**
Uganda	38	2	4	3	2	7	**56**
Zambia	6	1	0	1	0	1	**9**
Zimbabwe	0	0	0	1	0	0	**1**
East Africa, unspecified	11	0	0	0	0	2	13
West Africa, unspecified	8	0	2	2	0	0	12
Africa, unspecified	14	0	0	1	0	3	18
**Asia**	**5**	**91**	**2**	**4**	**2**	**19**	**123**
Afghanistan	0	15	0	3	0	2	**20**
Burma	0	1	0	0	0	0	**1**
Cambodia	0	1	0	0	0	1	**2**
India	3	48	1	1	2	9	**64**
Indonesia	1	1	0	0	0	0	**2**
Korea, South	0	1	0	0	0	1	**2**
Pakistan	0	23	0	0	0	4	**27**
Thailand	0	0	0	0	0	2	**2**
Vietnam	1	0	0	0	0	0	**1**
Southeast Asia, unspecified	0	1	1	0	0	0	2
**Central America and the Caribbean**	**39**	**16**	**0**	**0**	**0**	**11**	**66**
Dominican Republic	28	0	0	0	0	4	**32**
El Salvador	0	0	0	0	0	1	**1**
Guatemala	0	10	0	0	0	5	**15**
Haiti	11	0	0	0	0	0	**11**
Honduras	0	3	0	0	0	1	**4**
Mexico	0	1	0	0	0	0	**1**
Central America, unspecified	0	2	0	0	0	0	**2**
**South America**	**5**	**17**	**1**	**1**	**0**	**2**	**26**
Brazil	1	2	0	0	0	1	**4**
Colombia	1	1	0	0	0	0	**2**
Guyana	2	5	0	0	0	1	**8**
Peru	1	8	1	1	0	0	**11**
Venezuela	0	1	0	0	0	0	**1**
**Oceania**	**0**	**2**	**0**	**0**	**0**	**0**	**2**
Papua New Guinea	0	2	0	0	0	0	**2**
**Middle East**	**1**	**0**	**0**	**0**	**0**	**0**	**1**
Yemen	1	0	0	0	0	0	1
**Unknown**	**27**	**11**	**3**	**1**	**0**	**15**	**57**
**Total**	**1,011**	**176**	**62**	**45**	**12**	**179**	**1,485**

Of 165 imported *P. vivax* infections with information about region of acquisition, 91 (55.2%) were acquired from Asia, followed by 39 (23.6%) from Africa, 16 (9.7%) from Central America and the Caribbean, 17 (10.3%) from South America, and two (1.2%) from Oceania. In 2015, a total of 59 *P. ovale* and 44 *P. malariae* infections were reported with information about the region of acquisition; 56 (94.9%) and 39 (88.6%) were acquired in Africa, respectively. *P. vivax* cases imported from Central America and the Caribbean totaled 13 in 2014 and 16 in 2015.

### Region of Acquisition and Diagnosis

In 2015, the region of acquisition was known for 1,428 (96.2%) of 1,485 imported cases. Among these, 1,210 (84.7%) were acquired in Africa, 123 (8.6%) from Asia, and 66 (4.6%) from Central America and the Caribbean. Less than 2% of the imported malaria cases were acquired from South America (26 cases), Oceania (two cases), or the Middle East (one case).

Overall, 175 fewer cases were acquired from Africa in 2015 than in 2014 (Table 5). In 2015, most cases (781 [64.5%]) acquired from Africa were from the West Africa^§^ region, although this percentage decreased significantly in 2015 compared with 2014 (969 of 1,385 cases [70.0%]). The number of cases acquired from outside the West African region of Africa were approximately equivalent (416 in 2014 versus 429 in 2015), suggesting that the change in cases from West Africa accounts for the decrease in imported cases in 2015. The countries in West Africa that experienced widespread Ebola transmission during 2014–2015 (Guinea, Sierra Leone, and Liberia) collectively experienced a significant reduction of malaria cases in 2015: 148 of 781 (19.0%) cases versus 285 of 969 (29.4%) cases in 2014. Numbers of cases imported from Liberia were 125 in 2014 and 67 in 2015 and from Sierra Leone were 133 in 2014 and 41 in 2015. However, the number of cases from Guinea increased in 2015 compared with 2014 (40 versus 27 cases, respectively). Countries in Africa with more cases in 2015 include Côte d’Ivoire (31 more), Cameroon (20 more), Sudan (18 more), Guinea (13 more), Democratic Republic of the Congo (10 more), and Chad (9 more). Countries in Africa with fewer cases in 2015 include Sierra Leone (92 fewer), Liberia (58 fewer), Nigeria (56 fewer), Ethiopia (12 fewer) and Mali (9 fewer).

**TABLE 5:** Number of imported malaria cases and annual percentage change from 2014 to 2015, by region of acquisition — United States, 2014–2015

Area or region	2014	2015	Difference from 2014 to 2015	Annual percentage change from 2014 to 2015*
	No.	No.	No.	%
Africa (total)	1,385	1,210	-175	-87
Africa, West	969	781	-188	-93
Africa, other	416	429	13	6
Asia	160	123	-37	-18
Central America and the Caribbean	25	66	41	20
South America	29	26	-3	-1.5
Oceania	7	2	*-*5	*-*2.5
Middle East	1	1	0	0
Unknown	80	57	*-*23	*-*11
**Total**	**1,687**	**1,485**	**-202**	**—**

*Annual percentage change = [(Difference from 2015 – 2014)/202] X 100.

The number of cases imported from Asia in 2015 was not significantly different from the number imported in 2014 (123 [8.6%] and 160 [10.0%], respectively). India had the most cases imported from this region; cases from India decreased from 100 in 2014 to 64 in 2015. Cases imported from Afghanistan totaled 11 in 2014 and 20 in 2015, and the numbers of infections imported from Pakistan totaled 25 cases in 2014 and 27 cases in 2015.

Imported cases from Central and South America and the Caribbean increased significantly from 54 (3.4%) in 2014 to 92 (6.4%) in 2015. Although the number of cases imported from Central America (17 cases in 2014 and 23 cases in 2015) and South America (29 cases in 2014 and 26 cases in 2015) remained steady, in 2015 the number of cases imported from the Caribbean increased from eight in 2014 to 43 cases in 2015. Two cases were imported from the Dominican Republic in 2014, which increased to 32 in 2015. Of these 32 cases, 25 patients provided a reason for travel, and of these, 22 traveled to the Dominican Republic for tourism; three patients reported VFR travel. Seven cases described as a cluster were imported into Puerto Rico after patients went on a school trip and took no chemoprophylaxis (*36*,*37*). Although itinerary details are not systematically collected, nine of 32 persons from the Dominican Republic indicated that they had traveled to the resort area of Punta Cana. Five cases were imported from Haiti in 2014 and 11 in 2015; eight of 11 persons reported VFR travel, one each traveled as a missionary, for business, or as a refugee or an immigrant. Peru and Guyana contributed the majority of the 26 cases reported from South America (11 and eight cases, respectively). In Central America, cases imported from Guatemala increased from five in 2014 to 15 in 2015. Cases imported from Honduras decreased from eight in 2014 to four in 2015. In 2015, two cases were reported from countries in Central America with limited malaria transmission: one each from El Salvador and Mexico. One patient had symptom onset 4 days after returning from El Salvador for 1 week of VFR travel. The patient had an infection with an unknown species (confirmed by blood smear) and no parasitemia information. One person reported traveling to Mexico as a tourist for 10 days and returning to the United States 5 days before illness onset. The infection was confirmed by blood smear as *P. vivax* with 0.03% parasitemia. Neither case was confirmed by a public health laboratory or the CDC reference laboratory. The patients had symptoms 11 and 15 days after arrival to the area where malaria is endemic, suggesting short incubation periods. The patients were lost to follow-up, and no additional information could be obtained to confirm their travel histories.

**FIGURE 3:**
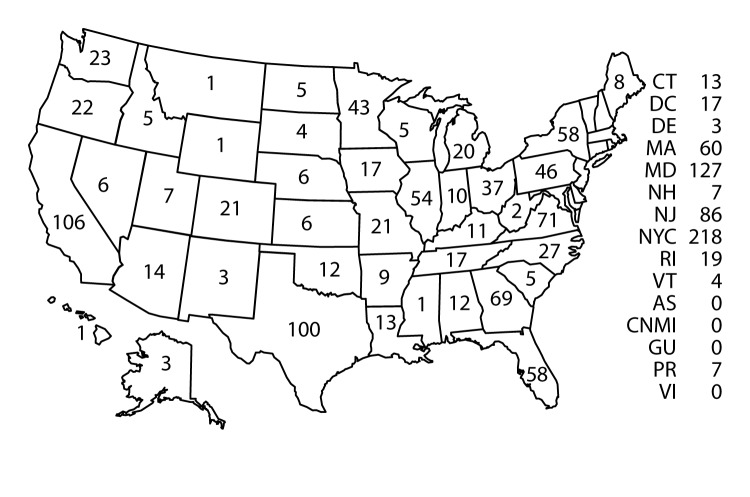
Number* of malaria cases, by state in which the disease was diagnosed — United States, 2015 **Abbreviations: **AS = American Samoa; CT = Connecticut; CNMI = Commonwealth of the Northern Mariana Islands; DC = Washington, DC; DE = Delaware; GU = Guam; MA = Massachusetts; MD = Maryland; NH = New Hampshire; NJ = New Jersey; NYC = New York City; PR = Puerto Rico; RI = Rhode Island; VI = Virgin Islands; VT = Vermont. * N = 1,517. California, Florida, Georgia, Illinois, Maryland, Massachusetts, New Jersey, New York City, New York state (not including New York City), Texas, and Virginia account for 66% of cases.

Confirmed cases were classified according to location of diagnosis or residence; 11 jurisdictions reported >50 cases of malaria in 2015, accounting for 66.4% of the 1,517 cases reported: New York City (218), Maryland (127), California (106), Texas (100), New Jersey (86), Virginia (71), Georgia (69), Massachusetts (60), Florida (58), New York State (not including New York City) (58), and Illinois (54) (Figure 3). Consistent with an overall reduction in the number of imported cases, most jurisdictions reported fewer malaria cases in 2015 compared with 2014. A small number of states reported more cases from 2014 to 2015, including California (96 and 106 cases [10.4% increase]), New Jersey (79 and 86 cases [8.9% increase]), and Puerto Rico (two and seven cases [250% increase], which had a cluster of cases in 2015. The jurisdictions with the most substantial percentage decrease in cases were in Pennsylvania (91 to 46 cases [49.5% decrease]), Washington (43 to 23 cases [46.5% decrease]), Arizona (23 to 14 cases [39.1% decrease]), North Carolina (41 to 27 cases [34.2% decrease]), Colorado (31 to 21 cases [32.3% decrease]), Georgia (83 to 69 cases [16.9% decrease]), and Maryland (144 to 127 cases [11.8% decrease]).

### Imported Malaria Among U.S. Residents and Nonresidents

Among 1,485 imported cases, 1,318 (88.8%) reported their residency status; 951 (72.2%) were U.S. residents and 367 (27.9%) were foreign residents (Table 6). The majority of infections among U.S. residents were acquired in Africa (823 [86.5%]), which was comparable to 2014 (996 [87.3%]) and continues a trend that has occurred since 2008 (*9*,*38*–*42*). Among U.S. residents, a significant decrease occurred in the number of imported cases acquired in Asia (84 [7.4%] in 2014 versus 47 [4.9%] in 2015). No significant change occurred from 2014 to 2015 in the number of U.S. residents with imported malaria infections from South America (19 [1.7%] in 2014 versus 18 [1.9%] in 2015). However, more than a twofold increase occurred in the number of imported malaria cases among U.S. residents from the Central America and Caribbean region (16 [1.4%] cases in 2014 versus 52 [5.5%] in 2015). The number of cases acquired among U.S. residents from Oceania remained <1.0% in 2014 and 2015 (seven [0.6%] in 2014 versus two [0.2%] in 2015).

**TABLE 6:** Number and percentage of imported malaria cases among U.S. and foreign residents, by region of acquisition — United States, 2015

Area or region	U.S. residents	Foreign residents	Total
	No. (%)	No. (%)	No. (%)
Africa	823 (86.5)	274 (74.7)	**1,097 (83.2)**
Asia	47 (4.9)	63 (17.2)	**110 (8.4)**
South America	18 (1.9)	7 (1.9)	**25 (1.9)**
Central America and the Caribbean	52 (5.5)	8 (2.2)	**60 (4.6)**
Oceania	2 (0.2)	0 (0)	**2 (0.2)**
Europe	0 (0)	0 (0)	**0 (0)**
Middle East	1 (0.1)	0 (0)	**1 (0.1)**
Unknown	8 (0.8)	15 (4.1)	**23 (1.8)**
**Total**	**951 (100)**	**367 (100)**	**1,318 (100)**

Three fourths of malaria cases imported among foreign residents in 2015 were from Africa (274 [74.7%]), followed by Asia (63 [17.2%]) and the Americas and Caribbean (15 [4.1%]). Among foreign residents, regional differences in the numbers of imported malaria cases did not vary significantly from 2014 to 2015. From 2014 to 2015, fewer cases were imported among residents of Ghana (30 to 15 cases), India (40 to 30 cases), Nigeria (75 to 67 cases), and Ethiopia (16 to eight cases). However, more cases were reported from 2014 to 2015 among residents of Côte d’Ivoire (10 to 17 cases), Democratic Republic of the Congo (11 to 15 cases), Afghanistan (eight to 15 cases), Burkina Faso (seven to 11 cases), and Sudan (one to seven cases).

In 2015, during the Ebola outbreak in West Africa, a significant decrease occurred in the proportion of U.S. residents who acquired malaria from West Africa: 710 (62.2%) in 2014 compared with 540 (56.8%) in 2015. Likewise, a twofold decrease occurred in the number of U.S. residents who acquired malaria from countries with widespread Ebola transmission (214 [18.7%] in 2014 versus 91 [9.6%] in 2015). The change from 2014 to 2015 in imported malaria cases among foreign residents from West Africa (194 [50.5%] in 2014 versus 175 [47.7%] in 2015) and among foreign residents from countries with widespread Ebola transmission (Ebola-affected countries) (51 [13.3%] in 2014 versus 41 [11.2%] in 2015) was not significant.

### Seasonality of Malaria Diagnosed in the United States

**FIGURE 4:**
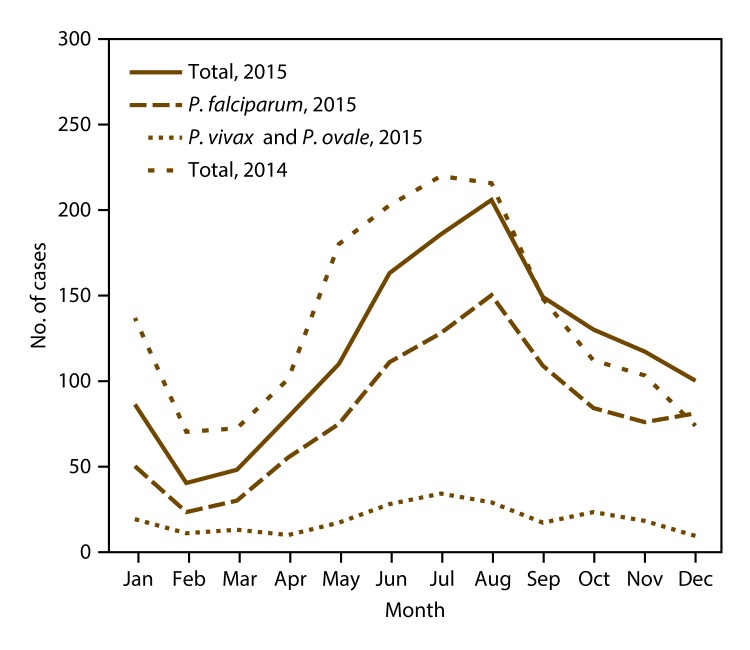
Number* of imported malaria cases, by *Plasmodium *species and month of symptom onset — United States, 2015 * Total number of cases for 2015 is 1,414, which includes the following: 216 *P. malariae,* mixed, and unknown species determination; 972 infections with *P. falciparum*; and 226 infections with *P. vivax* and *P. ovale*. Total number of cases for 2014 is 1,636 infections with all species.

As in previous years (*9*,*38*,*40*,*43*), imported malaria cases peaked during the summer holiday months of July and August 2015, with a mean of 196 imported cases in each month (Figure 4). A total of 208 fewer cases were reported in 2015 than in 2014. During January–July 2015, an average of 27.6% fewer cases of malaria were reported than during the same months in 2014. The peak numbers of cases in August of both years were comparable (216 in 2014 versus 206 in 2015), and during October–December 2015, the number of malaria cases reported was an average of 16.7% greater than it was during the same months in 2014. The lowest number of imported cases occurred during February and March, with an average of 44.0 cases during these months. *P. falciparum* species accounted for 150 (72.8%) of 206 cases imported in August 2015 and 23 (57.5%) of 40 cases in February 2015. *P. vivax* and *P. ovale* infections were combined because the illnesses have relapsing potential, and a secondary peak can occur after the main peak. In 2015, the mean number of *P. vivax* and *P. ovale* cases was 19.0 per month; the maximum (34 cases) occurred in July. The number of *P. vivax* and *P. ovale* cases was greater than the mean during June–August and in October. In 2015, no obvious secondary peak of imported cases for *P. vivax* and *P. ovale* occurred, although the numbers were small.

### Interval Between Arrival in the United States and Illness Onset

Among 1,306 imported cases with a *Plasmodium* species determination, 1,068 (81.8%) had complete information on return travel and illness onset dates, allowing for calculation of the interval between dates (Table 7). Among all patients, regardless of infecting species, 121 (11.3%) had symptom onset before arriving in the United States. Of 855 patients with *P. falciparum* infections, 818 (95.7%) had symptom onset before or within 29 days of arrival to the United States. In contrast, 51 (38.9%) of *P. vivax* patients and 25 (59.5%) of *P. ovale* patients had illness onset ≥30 days after arrival in the United States, consistent with the potential for these species to relapse because of the persistence of liver hypnozoites. Ninety-nine percent of infections of any species occurred within 1 year of arrival to the United States after travel to a country where malaria is endemic.

**TABLE 7:** Number and percentage of imported malaria cases, by interval between date of arrival in the United States and onset of illness and *Plasmodium* species* — United States, 2015

Interval (days)	*P. falciparum*	*P. vivax*	*P. ovale*	*P. malariae*	Mixed	Total
	No. (%)	No. (%)	No. (%)	No. (%)	No. (%)	No. (%)
<0^†^	99 (11.6)	16 (12.2)	3 (7.1)	2 (6.3)	1 (12.5)	**121 (11.3)**
0–29	719 (84.1)	64 (48.9)	14 (33.3)	16 (50.0)	4 (50.0)	**817 (76.5)**
30–89	25 (2.9)	22 (16.8)	10 (23.8)	14 (43.8)	1 (12.5)	**72 (6.7)**
90–179	9 (1.1)	10 (7.6)	6 (14.3)	0 (0)	0 (0)	**25 (2.3)**
180–364	1 (0.1)	15 (11.5)	7 (16.7)	0 (0)	2 (25.0)	**25 (2.4**)
≥365	2 (0.2)	4 (3.1)	2 (4.8)	0 (0)	0 (0)	**8 (0.8)**
**Total**	**855 (100)**	**131 (100)**	**42 (100)**	**32 (100)**	**8 (100)**	**1,068 (100)**

* Persons for whom *Plasmodium* species, date of arrival in the United States, or date of onset of illness is unknown are not included.

^†^ Cases in this row are in patients who had onset of illness before arriving in the United States.

### Imported Malaria Among U.S. Military Personnel

In 2015, a total of 23 cases of malaria diagnosed in the United States occurred among U.S. military personnel, comparable to the 31 cases reported in 2014 (1.5% and 1.8% of imported cases in 2015 and 2014, respectively). In 2015, a total of 16 cases of malaria among U.S. military members were acquired in Africa, of which nine were acquired in West Africa and three from Liberia during deployment for the Ebola response. Two cases were acquired from South Korea, two were from Afghanistan, and three did not have a reported country of acquisition. The numbers of cases acquired in Afghanistan were comparable in 2014 and 2015 (three and two cases, respectively). Sixteen (69.6%) of 23 cases among military personnel had a reported species: nine (56.3%) were *P. falciparum*, four (25.0%) were *P. vivax*, two (12.5%) were *P. ovale,* and one (6.3%) was *P. malariae*. Four of the six patients who were infected with a species capable of relapse (*P. vivax* or *P. ovale*) received primaquine to treat liver hypnozoites. Seventeen patients had information on chemoprophylaxis use; five did not take an antimalarial medication to prevent malaria. Of the 12 patients who took chemoprophylaxis, nine took an appropriate regimen for the region of travel, and five of these reported missing doses of the regimen. One patient had a severe illness after deployment in South Korea (severe anemia associated with *P. vivax* illness); no uniform policy exists requiring active-duty military personnel to take chemoprophylaxis during deployment in many parts of South Korea (*5*,*44*), and this patient did not take chemoprophylaxis. The only treatment reported was primaquine, and the patient recovered. 

Of the three military patients who acquired malaria during deployment to Liberia, all reported taking atovaquone-proguanil for chemoprophylaxis. One patient reported not missing any dose of chemoprophylaxis for a 5-week deployment during October–November 2014; he received the malaria diagnosis (confirmed by blood film microscopy) with an unknown species in February 2015. He was treated with mefloquine and recovered. Three months after his diagnosis and treatment, a convalescent blood sample was sent to CDC for reference laboratory testing. The PCR test of the convalescent sample was negative; however, serology results indicated reactivity to *P. malariae* antigens, with no reactivity to all other species examined, suggesting infection with *P. malariae*. A second military service member was deployed to Liberia for 6 months (dates unknown) and had symptom onset in mid-February 2015; in early March 2015, PCR testing confirmed a *P. ovale* infection*.* Neither treatment medications nor adherence to chemoprophylaxis was reported. A third member of the military was deployed to Liberia (dates unknown). He took atovaquone-proguanil for chemoprophylaxis but did not adhere to the regimen. He had symptom onset in April 2015, and a malaria diagnosis was confirmed by blood film microscopy in May 2015 (species unknown). He was treated with artemether-lumefantrine and recovered. 

### Chemoprophylaxis Use

Among U.S. residents, information about malaria chemoprophylaxis use was reported for 860 (90.4%) of 951 imported cases; of these patients, 632 (73.5%) indicated that no chemoprophylaxis regimen was taken during travel. Among the 228 patients who reported taking chemoprophylaxis, 49 (21.5%) did not indicate which medication was taken. Among 178 patients who reported specific drug information, 154 (86.5%) took a regimen recommended by CDC for the region of travel. Among 127 reports containing self-reported adherence information, 91 (71.7%) patients reported missing doses. Reported adherence to chemoprophylaxis in 2015 was 28.4% (36 of 127) and in 2014 was 38.1% (64 of 168). Among 154 patients who took CDC-recommended chemoprophylaxis, 53 (34.4%) took doxycycline, 49 (31.8%) took mefloquine, 45 (29.2%) took atovaquone-proguanil, six (3.9%) took two or more CDC-recommended medications, and one reported taking primaquine (0.7%); no patients took chloroquine alone. Among the 766 cases in U.S. residents with complete information on chemoprophylaxis, 36 (4.7%) patients were adherent to an appropriate regimen, 639 (83.4%) patients did not take chemoprophylaxis or took an incorrect regimen, and 91 (11.9%) patients did not adhere to an appropriate regimen. A total of 95.3% of U.S. residents with malaria did not adhere to or did not take a CDC-recommended chemoprophylaxis regimen.

#### Cases of *P. vivax* or *P. ovale* Infections After CDC-Recommended Prophylaxis Use

Primary prophylaxis can prevent acute illness in *P. vivax* and *P. ovale* infections, although the patient might have a relapse unless terminal prophylaxis with primaquine is taken to clear dormant hypnozoites (*26*). The infecting malaria species was known for 135 (87.7%) of 154 patients who took a CDC-recommended chemoprophylaxis regimen; *P. vivax* accounted for 11 (8.2%) and *P. ovale* for 13 (9.6%) of these cases. Sufficient information was available for 22 of the 24 *P. vivax* or *P. ovale* patients to calculate the number of days between the return travel date and the date of illness onset. Symptom onset occurred >45 days after return to the United States among 12 patients and is consistent with relapsing illness rather than failure of primary prophylaxis. Ten cases occurred ≤45 days after the patients returned to the United States, suggesting an acute infection and possible primary prophylaxis failure; five of the patients reported nonadherence to the chemoprophylaxis regimen, three reported missing no doses, and two did not provide information on adherence. Of the three patients with *P. vivax* or *P. ovale* infections who adhered to chemoprophylaxis, one traveled to Sudan for business for an unknown length of time, took atovaquone-proguanil for chemoprophylaxis, and became ill 30 days after return; one traveled to Peru for a 10-day missionary trip, took doxycycline for chemoprophylaxis, and became ill 12 days after return to the United States; and one spent 14 months in Togo as a missionary, reported taking mefloquine and doxycycline for chemoprophylaxis, and became ill 24 days after return. Potential reasons for infection in these patients include early relapse from hypnozoites established at the onset of travel (especially for the third case), inadequate dosing or malabsorption of the chemoprophylaxis medication, inaccurate reporting of adherence, or emerging parasite resistance.

#### *P. falciparum, P. malariae,* or Mixed Infections After CDC-Recommended Prophylaxis Use

Among the 135 patients with malaria with known species and who reported taking a CDC-recommended prophylaxis regimen, 99 (73.3%) of the infections were from *P. falciparum*, 11 (8.2%) were from *P. malariae*, and one (0.74%) was a mixed infection. Among the patients with *P. falciparum* infections, 97 (98.0%) reported travel to Africa, and 48 (48.5%) had traveled to West Africa. One *P. falciparum* case each occurred after persons traveled to Indonesia and the Dominican Republic. Of 85 *P. falciparum* case reports containing information on adherence to the recommended prophylaxis regimen, 65 (76.5%) patients indicated nonadherence. Twenty cases of *P. falciparum* occurred among patients who reported adherence to a recommended prophylaxis regimen; nine (45.0%) patients took mefloquine, seven (35.0%) took atovaquone-proguanil, and four (20.0%) took doxycycline. All 20 patients with *P. falciparum* infections who adhered to chemoprophylaxis acquired their infections in Africa, with nine (45.0%) from the West African region. Specimens from patients who adhered to the chemoprophylaxis regimen and had traveled to Uganda (two specimens) and Ghana (one specimen) were provided to CDC for molecular resistance surveillance; two patients reported taking doxycycline, and one took atovaquone-proguanil. No valid genetic markers are available to assess doxycycline resistance. The patient who reported adherence to atovaquone-proguanil had an atovaquone-sensitive genotype, suggesting that this marker for antimalarial resistance was not a factor. 

Eleven cases of *P. malariae* occurred after taking a recommended prophylaxis regimen. Among the five patients with information on adherence, one patient reported adhering to atovaquone-proguanil for chemoprophylaxis (as described in the previous section on malaria in military personnel). 

One patient acquired a mixed-species infection after taking a recommended prophylaxis regimen. This infection was PCR-confirmed, and the patient had traveled to Nigeria for an unknown duration, and was positive for *P. falciparum* and *P. ovale*. Mefloquine was reported as prophylaxis; however, no adherence information was provided, and the specimen was not available for molecular resistance testing. 

Possible explanations for *Plasmodium* infections in patients who adhered to chemoprophylaxis include inadequate dosing or malabsorption of the chemoprophylaxis medication, inaccurate reporting of adherence, or emerging parasite resistance.

### Patients with a Recent History of Malaria

Among the 1,485 cases of malaria imported into the United States in 2015, a total of 1,095 (73.7%) patients provided information on previous history of malaria; 202 (18.5%) reported having had malaria in the past 12 months. The infecting species for the previous illness was reported for 34 (16.8%) of 202 patients with a previous history of malaria; 19 (55.9%) recalled *P. falciparum*, eight (23.5%) recalled *P. vivax,* three (8.8%) recalled *P. ovale*, and four (11.8%) recalled *P. malariae* infection. Based on illness onset date, previous date of illness, and species, nine probable relapse cases were identified: eight caused by *P. vivax* and one caused by *P. ovale*. Two additional *P. ovale* cases were considered to be probable relapses, because the species was the same for the previous and current illnesses; however, the previous date of illness onset was missing. Among the 19 patients who recalled a *P. falciparum* infection in the past 12 months, 12 (63.2%) had a current infection with *P. falciparum.* Among the four patients who recalled a previous *P. malariae* infection in the past 12 months, three were infected with species other than *P. malariae,* and one was infected with an unspecified species.

### Reason for Travel

Among nonmilitary U.S. residents, the reason for travel to a country where malaria is endemic was reported for 753 (81.1%) cases of 928 imported malaria illnesses (Table 8). Of these, 529 (70.3%) case reports included VFR as the reason for travel; business travel was reported in 74 (9.8%); and missionary travel was reported in 63 (8.4%). Tourism was reported among 61 (8.1%) patients, an increase from 48 (5.5%) in 2014. Ten (1.3%) patients acquired malaria during travel for education (as students or teachers), which is a decrease from 2014, when 35 (4.0%) patients traveled for education. Two (0.3%) cases were related to Peace Corps service in 2015, and eight (0.9%) related cases were reported in 2014. Three cases occurred among airline or ship crew members (0.4%). Eleven patients provided other reasons for travel (1.5%).

**TABLE 8:** Number and percentage of imported malaria cases* among U.S. civilians and foreign residents, by purpose of travel at the time of acquisition — United States, 2015

Category	U.S. civilians	Foreign residents
	No. (%)	No. (%)
Visiting friends and relatives	529 (57.0)	82 (22.3)
Tourist	61 (6.6)	10 (2.7)
Missionary or dependent	63 (6.8)	6 (1.6)
Business	74 (8.0)	19 (5.2)
Student or teacher	10 (1.1)	15 (4.1)
Air crew or sailor	3 (0.3)	3 (0.8)
Peace Corps	2 (0.2)	0 (0)
Refugee or immigrant	0 (0)	120 (32.7)
Other	11 (1.2)	15 (4.1)
Unknown	175 (18.9)	97 (26.5)
**Total**	**928 (100)**	**367 (100)**

* N = 1,295.

Among the 367 imported cases among foreign residents, reason for travel was indicated for 270 (73.6%) cases (Table 8). Traveling to the United States as an immigrant or a refugee was cited in 120 case reports (44.4%). VFR travel to the United States from countries where malaria is endemic was reported by 82 (30.4%) patients, followed by 19 (7.0%) patients reporting business travel. Fifteen (5.6%) patients traveled for education (as a student or teacher) in 2015. Tourism was reported by 10 (3.7%) patients, missionary travel was reported by six (2.2%), and airline or ship crew travel was reported by three (1.1%) patients. Other reasons were reported by 15 (5.6%) patients. Of the 120 foreign residents who traveled to the United States as a refugee or an immigrant, 86 (71.7%) originated in Africa, followed by Asia (27 [22.5%]) and the Americas and Caribbean (five [4.2%]); two patients who were refugees or immigrants did not report their country of origin.

### Malaria by Age

Age was reported for 1,513 (99.7%) of 1,517 patients with malaria in 2015, and of these, adults aged ≥18 years accounted for 1,272 (84.1%) cases. Fifty-five (3.6%) patients were aged <5 years, and 95 (6.3%) were aged ≥65 years. Of the 241 children (aged <18 years), 103 (42.7%) were U.S. residents, and 88 (85.4%) of these had traveled to Africa. Among the U.S. civilian children, 78 (75.7%) traveled to visit friends and relatives, five (4.9%) traveled for missionary reasons, four (3.9%) for tourism, and one (1.0%) for education. No reason for travel was provided for 15 (14.6%) children. Among the 103 U.S. civilian children, information on chemoprophylaxis use and adherence was reported for 98 (95.1%); 67 (68.4%) did not take medication during travel to prevent malaria. Of the 31 children who took a chemoprophylaxis regimen, 22 (71.0%) took a CDC-recommended antimalarial that was appropriate for the region of travel. Of the 14 children with information on adherence, case reports from eight (57.1%) indicated that all doses were taken. All eight cases were acquired in Africa, seven were confirmed *P. falciparum*, two cases were severe, and all recovered from their illness.

### Hospitalization

In 2015, hospitalization information was reported for 1,362 (89.8%) of 1,517 confirmed cases; 981 (72.0%) persons were hospitalized for their malaria illness. A total of 714 (72.8%) of hospitalized persons had *P. falciparum* malaria, and 236 (24.1%) hospitalized persons had one or more signs or symptoms of severe malaria. No significant change in these percentages occurred from 2014 to 2015; in 2014, a total of 1,046 [70.0%] patients were hospitalized of 1,498 with complete information; 766 [73.2%] with *P. falciparum*; 257 [24.6%] with severe malaria).

### Treatment for Uncomplicated Malaria Cases

#### Overall

Among the 1,517 confirmed malaria cases in 2015, a total of 1,258 (82.9%) cases were uncomplicated malaria. Of these, 1,028 (81.7%) had antimalarial treatment information indicated on the case report form. The most common treatment administered for uncomplicated malaria in 2015 was atovaquone-proguanil, used to treat 609 (59.2%) of imported cases in patients with treatment information. Quinine-based treatment ranked second, with 159 (15.5%) patients treated with this regimen. Artemether-lumefantrine, the only artemisinin-based combination therapy approved by the Food and Drug Administration (FDA) for treatment of uncomplicated malaria in the United States, was administered to 119 (11.6%) patients. A total of 102 (9.9%) uncomplicated cases were treated with chloroquine, and 62 (6.0%) were treated with mefloquine. 

The *CDC Guidelines for Treatment of Malaria in the United States*, herein referred to as the CDC guidelines, provides recommendations for the treatment of malaria according to species, disease severity, and region of acquisition (*20*). Of the 1,028 imported uncomplicated cases with treatment information, 925 (90.0%) received treatment in accordance with CDC recommendations. The proportion of uncomplicated cases treated correctly in 2015 was 6.8 percentage points greater than that in 2014 (83.2%), a significant improvement. Of those treated correctly, 156 (16.9%) patients reported taking an additional antimalarial medication that exceeded what is recommended by the CDC guidelines. Among the 103 (10.0%) patients whose treatment was not in accordance with CDC recommendations, six (5.8%) were treated with the same antimalarial that the patient had used for chemoprophylaxis. To avoid potential toxicity and reduced efficacy, patients should not be administered the same antimalarial treatment that was used for chemoprophylaxis.

#### Adequacy of Treatment by Species

Among the 680 uncomplicated *P. falciparum* cases, 631 (92.8%) were treated according to CDC guidelines 109 (17.3%) of these patients received an antimalarial treatment that exceeded the recommendations. Three (6%) of 49 *P. falciparum* patients who were not treated in accordance with CDC recommendations were pregnant. Treatment was in accordance with CDC guidelines for 31 (93.9%) of 33 cases of uncomplicated *P. malariae,* and eight (25.8%) patients received an additional antimalarial medication. Of those with *P. vivax* and *P. ovale* infections, 116 (84.1%) and 39 (79.6%), respectively, were appropriately treated for their acute infection, of whom seven (6.0%) patients with *P. vivax* infections and three (7.7%) with *P. ovale* infections received an additional acute-phase antimalarial medication. Administration of primaquine to treat relapsing illness (the liver hypnozoite stage of the parasite) was reported for 58 (42.3%) of all 137 nonpregnant patients with *P. vivax* infections and treatment information and was administered to 46 (39.7%) of 116 nonpregnant patients with *P. vivax* infections who were appropriately treated for the acute-phase illness. Among the 48 nonpregnant patients with *P. ovale* infections, primaquine was administered to 18 (37.5%), and among the 39 nonpregnant patients with *P. ovale* infections appropriately treated for the acute-phase infection, primaquine was administered to 15 (38.5%). Five (55.6%) of nine mixed infections with treatment information recorded were treated according to CDC guidelines; three (60.0%) of these patients received an additional antimalarial. Malaria infections with an unknown species should be treated following recommendations for *P. falciparum* (*20*); 103 (86.6%) of 119 cases with unknown infecting species were treated appropriately; 26 (25.2%) of these were treated with an additional antimalarial for the acute illness. Of 118 nonpregnant patients with unknown infecting species, 21 (17.8%) were treated with primaquine.

### Severe Malaria

In 2015, a total of 259 (17.1%) of 1,517 cases were classified as severe cases. Eleven (4.3%) of the patients with severe malaria died. Among severe cases, 201 (77.6%) occurred in adults aged 18–64 years. However, children (aged <18 years) were significantly more likely to have severe malaria than adults (58 of 241 [24.1%] children aged <18 years versus 201 of 1,272 [15.8%] patients aged ≥18 years). Likewise, the proportion of persons aged ≥65 years with severe malaria was significantly higher than the proportion among patients aged <65 years (27 of 95 [28.4%] patients aged ≥65 years versus 232 of 1,418 [16.4%] patients aged <65 years). In 2015, a total of 78 (21.3%) of 367 foreign residents had severe malaria, compared with 172 (18.1%) of 952 U.S. residents; foreign residents were equally as likely as U.S. residents to have a severe illness. This finding varies from 2014, when foreign residents were significantly less likely to have severe illness than U.S. residents (58 [15.1%] versus 226 [19.7%], respectively). Among patients with severe malaria in 2015, a total of 219 (84.6%) had *P. falciparum* infections, 17 (6.6%) had infections with an unknown *Plasmodium* species, and 15 (5.8%) patients had *P. vivax* infections. In 2015, the species distribution among severe cases was similar to the distribution in 2014.

Among 230 (88.8% of 259 severe cases) patients with severe malaria and information on chemoprophylaxis, 52 (22.6%) reported having taken any medication for chemoprophylaxis; 16 (30.8%) of these did not provide adherence or chemoprophylaxis medication information. Of 32 patients with severe malaria with chemoprophylaxis adherence information, 25 (78.1%) did not adhere to or take a recommended regimen for malaria prevention, compared with 66.4% of persons with uncomplicated cases who did not adhere to chemoprophylaxis or take a correct regimen. Of the seven patients who adhered to a recommended chemoprophylaxis regimen, four took doxycycline, two took mefloquine, and one took atovaquone-proguanil. No specimens from these patients were tested at CDC for molecular resistance. Potential reasons for chemoprophylaxis failure in these patients with severe malaria include inadequate dosing or malabsorption of the chemoprophylaxis medication, inaccurate reporting of adherence, or emerging parasite resistance.

Patients with severe malaria can have multiple clinical complications; acute kidney injury was most common and was experienced by 37 (14.3%) patients. Cerebral malaria was reported for 30 (11.6%) patients, acute respiratory distress syndrome for 28 (10.8%), severe anemia for 26 (10.0%), and jaundice for 12 (4.6%). Case reports from 198 patients with severe malaria contained information on the percentage of *Plasmodium* species parasitemia, of which 146 (73.7%) had ≥5% parasitized red blood cells. CDC guidelines state that patients with severe malaria should be treated aggressively in an inpatient setting with intravenous quinidine gluconate or artesunate. In 2015, a total of 18 (7.0%) patients with severe malaria were not hospitalized; the hospitalization status for five patients (1.9%) was unknown. Quinidine gluconate is the only FDA-approved medication for severe malaria. Artesunate is not FDA approved but is available from CDC as an investigational new drug for patients who cannot tolerate quinidine gluconate or when it is not available (*45*). In 2015, a total of 144 (55.6%) patients with severe malaria were treated with quinidine gluconate, and 54 (20.9%) were treated with artesunate. A total of 173 (66.8%) patients with severe malaria were treated with a parenteral antimalarial regimen, and 74 (28.6%) were treated with an oral regimen alone; treatment information was unknown for 12 (4.6%) patients. Among the 11 patients who died, three received both quinidine gluconate and artesunate, three received quinidine gluconate, one received artesunate, and one received an oral antimalarial treatment; three patients received no antimalarial treatment because they did not seek care and received the malaria diagnosis after being found unresponsive or dead. Among 32 pregnant women, 12 had severe malaria, of whom nine received parenteral treatment; five received quinidine gluconate, three received artesunate, and one received both parenteral treatments. Two pregnant women with severe malaria received oral antimalarial treatment, and treatment was unknown for another. Appropriate treatment of severe malaria improved significantly from 2014 to 2015. In 2014, a total of 138 (49.3%) of 280 patients received appropriate treatment, compared with 147 (59.3%) of 248 patients in 2015. However, patients with severe malaria were significantly more likely to have inappropriate treatment (101 [40.7%] of 248) than patients with uncomplicated malaria (103 [10.0%] of 1,028.

Although all malaria *Plasmodium* species can cause severe malaria, the rapid proliferation of *P. falciparum* parasites in nonimmune persons requires timely diagnosis and treatment to prevent progression to hyperparasitemia and severe illness. In 2015, on average, inpatients with *P. falciparum* infections had been hospitalized on day 4 after illness onset, regardless of disease severity (with a mean of day 4.2 for uncomplicated cases and day 4.3 for severe cases). Because the malaria case report form does not include the date of the clinical visit for patients who are not hospitalized, determining the time between illness onset and when outpatients seek medical care is not possible.

Among the 259 patients with severe illness, 234 (90.4%) infections were acquired in Africa; 17 (6.6%) in Asia; <1% in the Caribbean (two cases), Central America (two cases), or Oceania (one case); three severe cases were acquired in an unknown region. Among the 218 patients with severe malaria who reported a reason for travel, 122 (56.0%) reported VFR as the primary reason travel, of whom 89 (73.0%) had traveled to West Africa. Twenty-seven (12.4%) patients with severe malaria traveled to the United States as immigrants or refugees, or for business; 17 (7.8%) patients traveled for missionary work. Three patients (1.4%) with severe malaria traveled for education (as a student or teacher), one was an airline or a ship crewmember, and one was a member of the U.S. military. A total of 47 patients had unknown (41 [15.8%]) or other reasons for travel (six [2.3%]).

### Malaria During Pregnancy

Among 546 women with malaria diagnosed in the United States in 2015, a total of 32 (5.9%) were pregnant, and 12 (37.5%) of these had severe malaria. Twenty-seven (84.4%) pregnant women were hospitalized, including 12 patients with severe malaria, and all recovered. One woman with a PCR-confirmed *P. ovale* infection with <1% parasitemia also had hyperemesis gravidarum; she was treated with parenteral medication. She initially received quinidine gluconate, which was discontinued after detection of a prolonged heart-rate corrected Q-T interval (QTc). She then received artesunate and recovered. Although systematic information was not collected on birth outcomes, clinical notes were available for five pregnant women, indicating that delivery occurred near the time of infection. Of these, two infants were healthy. One infant had congenital malaria, and one was born at 27 weeks’ gestation and treated in the neonatal intensive care unit. One pregnant woman, originally from Tanzania, experienced fever and had a low platelet count at 31 weeks’ gestation. These symptoms were interpreted as signs of hemolysis, elevated liver enzymes, and low platelets (HELLP) syndrome, and an emergency cesarean section was performed 2 days after symptom onset. The woman continued to have fever and a low platelet count, and 3 days after delivery (5 days after illness onset), she was tested for malaria and found to have a *P. falciparum* infection with 16% parasitemia. The woman was treated with artesunate and atovaquone-proguanil and recovered. Her infant tested negative for malaria.

Twenty-three (71.9%) pregnant women had *P. falciparum* infections, six (18.8%) were infected with an unspecified *Plasmodium* species, two (6.3%) had infections with *P. ovale,* and one with *P. vivax*. Most pregnant women (21 [65.6%]) were foreign residents, and 11 (34.4%) infections were in U.S. residents. Of 25 (78.1%) women who indicated a reason for travel, 14 (56.0%) were VFR travelers, six (24.0%) were refugees or immigrants, and one each (4.0%) traveled as a missionary or for education (as a student or a teacher) or tourism. Two (8.0%) women provided other reasons.

Antimalarial drug choices to prevent or treat malaria during pregnancy are limited. In most areas where malaria is endemic, only mefloquine is approved for chemoprophylaxis; mefloquine and quinine with clindamycin are recommended as treatment for uncomplicated malaria during pregnancy (*20*). Pregnant women can use chloroquine for preventive chemoprophylaxis or for the treatment of uncomplicated malaria acquired from areas with chloroquine-sensitive malaria. Primaquine can cause hemolytic anemia among persons with glucose-6-phosphate dehydrogenase (G6PD) deficiency and should not be administered during pregnancy. Severe malaria in pregnant women is considered life threatening and should be treated aggressively with regimens including parenteral quinidine-gluconate or artesunate. Nine (81.8%) of 11 pregnant U.S. residents provided information on chemoprophylaxis use: eight women did not take chemoprophylaxis to prevent malaria during travel, and one took an unknown medication (to which she did not adhere) that was obtained while traveling in Africa. Treatment information was available for 27 (84.4%) pregnant women with malaria. Of these, 9 (33.3%) were not treated appropriately for malaria, and one (3.7%) patient received an antimalarial medication that is appropriate for malaria but is not FDA approved for use during pregnancy (artemether-lumefantrine). No pregnant women were treated with primaquine.

### Drug Resistance Markers

In 2015, CDC received 209 whole blood samples for molecular resistance monitoring. Of these, 56 samples were from patients who did not have *P. falciparum* infections, and no resistance monitoring was conducted. Molecular resistance surveillance was performed on 151 *P. falciparum* samples and two mixed-infection samples (one each *P. falciparum* with *P. vivax,* and *P. falciparum* with *P. ovale)* (Table 9). One jurisdiction submitted 55 anonymized samples that could not be matched epidemiologically to cases for regional analysis, and 12 samples were epidemiologically matched but did not have travel history information (Table 9). Of the 153 *P. falciparum* samples tested, 132 (86.3%) contained polymorphisms associated with pyrimethamine resistance, and 115 (87.1%) of these had three or more pyrimethamine resistance markers. One sample was not amplified for sulfadoxine resistance analysis, and 112 (73.7%) had at least one marker associated with sulfadoxine resistance; 13 (11.6%) of these had three or more sulfadoxine resistance markers. Of 153 samples tested for chloroquine resistance markers, 105 (68.6%) did not have the resistant genotype. Amplification of the mefloquine locus was completed for 141 of 153 samples, and six (4.3%) contained the resistant genotype. Amplification of the genes associated with atovaquone resistance was possible for 135 of 153 samples, and none had the polymorphism associated with the resistance. Of 149 samples that could be amplified for artemisinin resistance, one sample had a marker associated with resistance. Because of widespread resistance to pyrimethamine and sulfadoxine, CDC does not recommend using drugs containing these components to treat malaria in the United States (*20*). None of the patients with chloroquine resistance markers and a reported travel history had exposure to malaria in a chloroquine-sensitive region. Five of six patients with markers of mefloquine resistance had traveled to Africa (Ghana, Guinea, Mozambique, Nigeria, and Uganda); one did not report the region of travel. Of these six, three reported taking chemoprophylaxis, one took atovaquone-proguanil and was adherent, and two patients reported taking mefloquine but did not adhere to the regimen. The two cases in these patients might have resulted from induced mefloquine resistance. No patients with a mefloquine resistance genotype were treated with mefloquine, and all recovered from their illness. CDC testing detected the K13 polymorphism associated with artemisinin resistance (*31*) in a sample from one patient who had traveled to Ghana and took no prophylaxis; this patient was not treated with an artemisinin-based therapy and recovered.

**TABLE 9:** Antimalarial drug resistance marker results among *Plasmodium falciparum* specimens, by drug and region of malaria acquisition — United States, 2015

Resistance markers	Area or region							
	Africa	Asia	Central America and the Caribbean	South America	Oceania	Middle East	Unknown	Total
	No. (%)	No. (%)	No. (%)	No. (%)	No. (%)	No. (%)	No. (%)	No. (%)
**Pyrimethamine**	**79 (51.6)**	**0 (0)**	**7 (4.6)**	**0 (0)**	**0 (0)**	**0 (0)**	**67 (43.8)**	**153 (100)**
No resistance markers	5 (3.3)	0 (0)	7 (4.6)	0 (0)	0 (0)	0 (0)	9 (5.9)	21 (13.7)
1 resistance marker	2 (1.3)	0 (0)	0 (0)	0 (0)	0 (0)	0 (0)	3 (2.0)	**5 (3.3)**
2 resistance markers	8 (5.2)	0 (0)	0 (0)	0 (0)	0 (0)	0 (0)	4 (2.6)	**12 (7.8)**
≥3 resistance markers	64 (41.8)	0 (0)	0 (0)	0 (0)	0 (0)	0 (0)	51 (33.3)	**115 (75.2)**
**Sulfadoxine**	**78 (51.3)**	**0 (0)**	**7 (4.6)**	**0 (0)**	**0 (0)**	**0 (0)**	**67 (44.1)**	**152 (100)**
No resistance markers	18 (11.8)	0 (0)	0 (0)	0 (0)	0 (0)	0 (0)	22 (14.5)	**40 (26.3)**
1 resistance marker	38 (25.0)	0 (0)	7 (4.6)	0 (0)	0 (0)	0 (0)	21 (13.8)	**66 (43.4)**
2 resistance markers	13 (8.6)	0 (0)	0 (0)	0 (0)	0 (0)	0 (0)	20 (13.2)	**33 (21.7)**
≥3 resistance markers	9 (5.9)	0 (0)	0 (0)	0 (0)	0 (0)	0 (0)	4 (2.6)	**13 (8.6)**
**Chloroquine**	**79 (51.6)**	**0 (0)**	**7 (4.6)**	**0 (0)**	**0 (0)**	**0 (0)**	**67 (43.8)**	**153 (100)**
No resistance markers	50 (32.7)	0 (0)	7 (4.6)	0 (0)	0 (0)	0 (0)	48 (31.4)	**105 (68.6)**
1 or 2 resistance markers	0 (0)	0 (0)	0 (0)	0 (0)	0 (0)	0 (0)	0 (0)	**0 (0)**
≥3 resistance markers	29 (19.0)	0 (0)	0 (0)	0 (0)	0 (0)	0 (0)	19 (12.4)	**48 (31.4)**
**Mefloquine**	**70 (49.7)**	**0 (0)**	**6 (4.3)**	**0 (0)**	**0 (0)**	**0 (0)**	**65 (46.1)**	**141 (100)**
No resistance markers	65 (46.1)	0 (0)	6 (4.3)	0 (0)	0 (0)	0 (0)	64 (45.4)	**135 (95.7)**
1 resistance marker	5 (3.6)	0 (0)	0 (0)	0 (0)	0 (0)	0 (0)	1 (0.7)	**6 (4.3)**
**Atovaquone**	**66 (48.9)**	**0 (0)**	**5 (3.7)**	**0 (0)**	**0 (0)**	**0 (0)**	**64 (47.4)**	**135 (100)**
No resistance markers	66 (48.9)	0 (0)	5 (3.7)	0 (0)	0 (0)	0 (0)	64 (47.4)	**135 (100)**
1 resistance marker	0 (0)	0 (0)	0 (0)	0 (0)	0 (0)	0 (0)	0 (0)	**0 (0)**
**Artemisinin**	**78 (52.4)**	**0 (0)**	**5 (3.4)**	**0 (0)**	**0 (0)**	**0 (0)**	**66 (44.3)**	**149 (100)**
No resistance markers	77 (51.7)	0 (0)	5 (3.4)	0 (0)	0 (0)	0 (0)	66 (44.3)	**148 (99.3)**
1 resistance marker	1 (0.7)	0 (0)	0 (0)	0 (0)	0 (0)	0 (0)	0 (0)	**1 (0.7)**

### Selected Malaria Case Reports

#### Congenital Case

One case of congenital malaria was reported in 2015. Parasites were transmitted from mother to child during pregnancy or during labor and delivery.

**Case 1.** A pregnant women aged 26 years immigrated to the United States in December 2014 from Pakistan. She gave birth in early June 2015 at 37 weeks gestation and experienced a fever a few days later. She received a diagnosis of a *P. vivax* malaria infection with 0.3% parasitemia and was treated with quinine. Blood smear microscopy was performed on a sample from the infant and was negative for malaria. Three weeks later, the infant experienced fever and received a diagnosis a *P. vivax* malaria infection with 2.2% parasitemia, thrombocytopenia, anemia (hemoglobin 7.9 g/dL), and splenomegaly. The infant received intravenous quinidine, clindamycin, and platelet transfusions. After 6 days of inpatient care, the infant recovered and was discharged.

#### Fatal Cases

Eleven fatal malaria cases were reported in 2015.

**Case 1**. A man aged 25 years spent 6 months teaching in Ghana. For 2 months he took atovaquone-proguanil for malaria chemoprophylaxis but reportedly stopped early because he thought it was unnecessary. His symptoms began on February 27, 2015, a total of 3 days after returning to the United States, and he was hospitalized on March 1, 2015; he received a diagnosis of cerebral malaria from a *P. falciparum* infection with 29% parasitemia. He was treated with intravenous quinidine and doxycycline, and in the early afternoon of March 2, 2015, he experienced two cardiac arrests. After the second episode, he was nonresponsive. Artesunate was obtained from CDC; the first dose was administered at 8:30 p.m. on March 2, 2015, and the parasitemia rapidly resolved. Approximately 2 hours after administration of artesunate, electroencephalography demonstrated an absence of significant brain activity. Subsequent imaging and perfusion studies demonstrated cerebral herniation and brain death. The patient was pronounced dead on March 15, 2015.

**Case 2.** In November 2014, a woman aged 69 years traveled to India for 4 weeks, returning on December 8, 2014. On December 11, 2014, she experienced a fever and headache. On December 21, 2014, she was admitted to the hospital with a diagnosis of malaria caused by *P. vivax* and was treated with quinine and doxycycline. She developed acute respiratory distress syndrome and remained hospitalized for 3 weeks before returning home. She was not treated with primaquine to prevent relapses. She remained in the United States and did not travel out of the country again. On November 11, 2015, her fever returned. On November 21, she received a diagnosis of an uncomplicated *P. vivax* malaria infection and was hospitalized. She was treated with quinine, doxycycline, and primaquine. Her fever resolved, and she appeared to be clinically stable. The patient was discharged from the hospital on November 23 but then died on November 25. No autopsy was performed, and no additional investigation occurred.

**Cases 3 and 4.** Two men aged 45 and 44 years who were residents of an African country where malaria is endemic had recently traveled for work to one or two other countries in Africa where malaria is endemic before traveling to the United States for a vacation. On May 1, 2015, they arrived in Chicago, Illinois, after traveling through Amsterdam, Netherlands. According to law enforcement officials and public health investigation records, the man aged 45 years was the first to feel ill, with onset of headache and body aches on May 1 while in Amsterdam. The man aged 44 years reported similar symptoms on May 2. The men were found dead in adjacent hotel rooms on May 9, 2015. The county medical examiner performed autopsies and sent tissue samples from multiple organs from both men to the CDC reference laboratory, which confirmed *P. falciparum* infections in both patients based on immunohistochemistry and PCR. Parasites from the men contained identical molecular resistance signatures, suggesting that the infections were acquired from the same region. The parasites contained the triple mutation in the *dhfr* gene and a single mutation in the *dhps* gene (pyrimethamine and sulfadoxine resistance markers), which are a common signature of parasites from Africa. However, microsatellite analysis revealed discrepant signatures at seven loci, and the man aged 45 years was infected with two different parasite strains. These data suggest that the men acquired the infections independently. No drug paraphernalia were found in either hotel room, nor was there evidence of a struggle or any disarray. No weapons or poisons were found. Law enforcement officials did not suspect foul play in their deaths.

**Case 5.** A woman aged 67 years from India who had diabetes and a recent history of a myocardial infarction visited the United States. Two days after arrival in the United States, she experienced fever, and 5 days later, she sought medical attention, with a temperature of 102.2°F (39°C). A malaria antigen test was negative; the malaria smear was positive, indicating a *P. vivax* infection with 1.8% parasitemia, which was confirmed by the state public health laboratory. The woman had lactic acidosis and hypotension and was admitted to the hospital intensive care unit. She was treated with intravenous quinidine; electrocardiography did not reveal QTc prolongation or QRS widening. On the second day in the hospital, the woman experienced a cardiac arrest and died.

**Case 6.** In July 2015, a man aged 64 years returned from a 3-month visit in Ghana to visit friends and relatives, and he did not take chemoprophylaxis. Symptoms began 8 days before his return to the United States. Two days after his return, he was admitted to the hospital with hypotension, renal failure, and respiratory distress caused by a severe *P. falciparum* infection with 20% parasitemia. Health care providers initially treated the man with intravenous quinidine and doxycycline; however, after QTc prolongation was detected, artesunate was obtained from CDC. The patient experienced a cardiac arrest but was resuscitated. He received his first and only dose of artesunate a few minutes after resuscitation. A few hours later, he experienced a second cardiac arrest and died.

**Case 7.** In 2012, a woman aged 25 years from Ghana with a history of sickle cell disease moved to the United States. In late August 2015, she returned from visiting friends and family in Ghana, and she did not take chemoprophylaxis. Symptoms began on September 5 with a headache and fatigue. On September 7, she had yellow eyes and called her physician; however, the office was closed for Labor Day, so she sought treatment on the morning of September 8; after consultation at the primary care clinic, her health care provider called an ambulance. In the emergency department, the initial diagnosis was sickle cell crisis. Laboratory tests revealed an increased anion gap (22 mEq/L), lactic acidosis, severe hemolytic anemia (hemoglobin 4.1 g/dL and bilirubin 40 mg/dL), and the blood smear was positive for a *P. falciparum* infection (2% parasitemia). The spleen was not visualized with abdominal ultrasound. She was admitted and administered oral artemether-lumefantrine. Later the same day, the woman became confused and agitated and then unresponsive. She was noted to be in asystole and was resuscitated twice in the next few hours. She was treated with blood transfusions and intravenous bicarbonate, quinidine, and doxycycline. The woman became progressively more acidotic and died approximately 14 hours after arriving at the hospital.

**Case 8.** A man aged 62 years traveled to Ghana for 1 month to visit friends and relatives. He did not take chemoprophylaxis. One day before returning to the United States, he experienced fever, sweating, myalgia, nausea, vomiting, and diarrhea. He also noticed that his eyes were turning yellow. He did not seek medical attention. Five days after returning to the United States, he attempted to return to work but was sent home because he was visibly ill. Three days later, he was found dead at his home. An autopsy revealed that he had a *P. falciparum* infection with parasites in multiple organs. The species was confirmed using immunohistochemical staining specific for *P. falciparum*.

**Case 9.** A man aged 58 years from Egypt visited the United States after a 2-week business trip in Nigeria. On January 27, 2015, while in the United States, he experienced fever and a headache and immediately sought medical attention. Initial laboratory tests were unrevealing. Tests were repeated approximately 12 hours later and indicated acute kidney injury and metabolic acidosis. A blood smear was positive for malaria parasites at 4 a.m. on January 28, 2015, with 80% parasitemia. The man was treated with oral quinine and doxycycline. He became confused and somnolent. Because quinidine was not available, CDC provided artesunate. Before administration of the first dose of artesunate, computed tomography (CT) of the head demonstrated cerebral edema in the posterior fossa. The man received all 4 doses of artesunate and an exchange transfusion, and the parasitemia resolved. He remained unresponsive and hospitalized in the intensive care unit, receiving mechanical ventilation and dialysis until March 13, 2015, when he was pronounced dead.

**Case 10.** A man aged 63 years traveled from the United States to Tanzania for a hunting trip and took no chemoprophylaxis. He returned to the United States on September 28, 2015, and was found unresponsive on the morning of October 5. When he arrived at the hospital, he received a diagnosis of severe cerebral malaria and lactic acidosis from a *P. falciparum* infection with 23.3% parasitemia. He received intravenous quinidine gluconate; however, because a sufficient amount was not available, artesunate was obtained from CDC. The man received the first of 4 doses of artesunate on the evening of October 5, and the subsequent treatment included doxycycline as recommended, resulting in the resolution of the parasitemia. The patient remained comatose throughout his hospitalization. CT of the head on October 9 demonstrated severe cerebral ischemia. A decision was made to withdraw support, and the patient died on October 12.

**Case 11.** A woman aged 75 years visited India for 6 months during June 2014-January 2015 but did not take malaria chemoprophylaxis. She had onset of fever and abdominal pain on May 28. She sought care in the emergency department on May 30 but initially denied any foreign travel; therefore, no malaria testing was performed. She returned on June 1 as her symptoms continued and was discharged again without evaluation for malaria. On June 5, she returned with worsening symptoms including shortness of breath and confusion. At this time, she had lactic acidosis and thrombocytopenia, and a malaria blood smear was positive with a 2% parasitemia for unspecified *Plasmodium* species. Her initial antimalarial treatment included atovaquone-proguanil, which was changed to intravenous quinidine with doxycycline. Her shortness of breath progressed as she developed acute respiratory distress syndrome necessitating intubation and mechanical ventilation. She also developed acute kidney injury requiring dialysis. Despite resolution of her parasitemia, she continued to experience multisystem organ failure, and life support was withdrawn on June 10. She died on the same day.

## Discussion

Although malaria cases diagnosed in the United States have generally been increasing since the 1970s, 208 fewer cases of malaria were reported in the United States in 2015 than in 2014 (*46*). During 2010–2014, an average of 1,754 cases per year were reported, and the total in 2015 is the lowest since 2009. Significantly fewer cases were acquired from West Africa in 2015 than in 2014, although no other significant regional decreases occurred. During January–July 2015, an average of 27.6% fewer imported malaria cases were diagnosed in the United States than during the same months in 2014. However, during the last quarter of 2015, an average of 16.7% more cases were reported than in the same period in 2014. These regional and temporal patterns suggest that the decreased number of imported malaria cases could be associated with changes in travel related to the Ebola outbreak in West Africa. WHO declared Ebola in West Africa to be a public health emergency of international concern on August 8, 2014, and provided recommendations for emergency management, including guidance for screening persons traveling at international airports (*33*). CDC issued travel warnings (*47*) and in collaboration with U.S. Customs and Border Protection initiated enhanced risk assessment and management procedures for travelers from the Ebola-affected countries of Guinea, Sierra Leone, and Liberia (*34*). Collectively, malaria cases imported into the United States in 2015 from the Ebola-affected countries decreased by 6.8 percentage points. However, malaria cases from Guinea were higher in 2015 than in 2014. The United Nations World Tourism Organization (UNWTO) reported tourist arrival data for two of three Ebola-affected countries. Whereas tourist arrivals in Guinea increased by 6.1% from 2014 to 2015, they decreased in Sierra Leone by 45.6% (*48*). According to situational reports, none of the Ebola-affected countries had widespread transmission of Ebola during the last quarter of 2015, although additional cases continued to occur into 2016 (*49*–*52*).

Malaria is endemic in all Ebola-affected countries, and febrile illnesses such as malaria and Ebola can be clinically indistinguishable, especially early in the course of the illness. To prevent potentially life-threatening complications, a malaria assessment and treatment should not be delayed for persons being tested for Ebola (*53*). In response to the West Africa Ebola outbreak, CDC developed additional steps to inactivate viruses, including Ebola, during the malaria blood slide preparation process (*25*). During October 2014–December 2015, state and local public health authorities actively monitored travelers from Ebola-affected countries for 21 days after arrival in the United States to detect fevers (*54*). CDC recommended that infection prevention and control precautions be used for persons with fever who had traveled to an Ebola-affected country until Ebola virus was ruled out or until an alternative diagnosis was established (*55*).

CDC and public health authorities investigated patients with confirmed malaria cases who initially stated they had not traveled outside the United States or provided an inaccurate statement and were then determined to have traveled to an Ebola-affected country or West Africa. Health care providers should always be aware of the possibility that patients might not fully or accurately disclose their travel history, especially during a public health emergency such as an Ebola epidemic.

The proportion of *P. falciparum* infections (67.4%) in 2015 is comparable to that in 2014 (66.1%); these percentages are the highest recorded for the period of 2005–2015. Use of PCR testing to identify the *Plasmodium* species might have contribute to this finding. Travel to the African region also could contribute to the large proportion of *P. falciparum* infections; UNWTO reports an overall in increase in travel rates to sub-Saharan Africa, with 35.4 million international tourist arrivals reported for 2015, an increase from 34.6 million in 2014 (*48*). Likewise, flights from the United States to Africa among U.S. citizens increased by approximately 12,000 per year since 1996 (*35*). An analysis of the GeoSentinel global surveillance network, which monitors travel-related illness, showed that 83% of travelers who contracted malaria were exposed in sub-Saharan Africa (*56*). Likewise, a meta-analysis with data from 2005–2015 showed that among imported malaria infections in 40 countries where malaria is not endemic, 56% of cases were from West Africa (*57*).

Chemoprophylaxis is the most effective way for U.S. residents to prevent malaria during travel to a country where malaria is endemic; however, persons who receive a diagnosis of malaria in the United States often do not obtain or adhere to chemoprophylaxis. Barriers to adherence include lack of awareness about the disease and its potential severity (*58*–*61*) and fear of adverse effects (*61*). International travelers are heterogeneous, with different motivations, levels of education, and barriers to chemoprophylaxis use. They include both short-stay travelers (e.g., tourists and airline crew members) and long-term travelers (e.g., Peace Corps volunteers, missionaries, disaster and relief workers, and military personnel). Chemoprophylaxis regimens must be tailored according to age, pregnancy status, destination country, patient preferences (e.g., daily or weekly drug regimen), and tolerability of potential side effects (e.g., sun sensitivity for doxycycline and neuropsychiatric disorders for mefloquine). However, persons who are properly educated about the risk for malaria illness and the safety and effectiveness of malaria chemoprophylaxis can become motivated to take the medication correctly for the duration of their exposure (*62*–*64*). Malaria importation among U.S. residents who visit friends and relatives is common (57.0%), and only 21.2% of cases imported into the United States among VFR travelers in 2015 reported any chemoprophylaxis use. Chemoprophylaxis use among children (persons aged <18 years) and adherence is largely determined by parents or guardians; 68.4% of children did not take medication to prevent malaria while traveling in countries where malaria is endemic. Health care providers should talk to their patients, especially VFR travelers who might travel to countries where malaria is endemic, about upcoming travel plans and offer education and medication to prevent malaria. Resources for discussing malaria prevention among VFR travelers is available on the CDC website (https://www.cdc.gov/malaria/travelers/vfr.html).

UNWTO estimates that during 2010–2030, tourist arrivals will increase at a rate of 4.4% per year in emerging destinations, including Africa, compared with a 2.2% increase in traditional destinations like Europe and North America (*48*). As international travel increases, prevention strategies and health communication messages become even more important for protecting travelers from infectious diseases and reducing risk for importation of acquired illnesses that are a threat for continual transmission in the United States. Malaria prevention should be emphasized before typical travel seasons in late spring and early summer, accompanied with reminders in late fall through early winter. Travelers should be informed of the risk for malaria and encouraged to use protective measures, especially chemoprophylaxis.

The best method for immediate diagnosis of malaria is microscopy. However, PCR testing is particularly valuable for species confirmation and should be used to confirm the results of microscopy and to evaluate for mixed infections. In 2014, the Council of State and Territorial Epidemiologists released a revised malaria case definition highlighting the importance of determining the species and the parasitemia percentage at the time of diagnosis and encouraging PCR testing for each case (*15*). Thick blood smears are more sensitive for detecting malaria parasites because the blood is concentrated, allowing for a greater volume of blood to be examined. Thin smears facilitate identification and quantification of the parasite species (*65*). Blood smears should be read immediately, and the percentage of parasitized red blood cells should be calculated and provided promptly to the treating clinician; qualified personnel who can perform this task should be on call after hours. Laboratories that can perform a complete blood count with a manual differential have the resources to do a malaria blood smear. CDC provides telediagnosis assistance to laboratories and care providers who need help promptly reading the blood smears (*66*). A 2010 nationwide survey of laboratories in the United States showed that most laboratories offered malaria diagnostic testing services, although very few were in complete compliance with all of the Clinical and Laboratory Standards Institute guidelines for analysis and reporting of results (*67*). In addition, most laboratories reported very few cases annually (*68*). Laboratories unable to provide immediate blood film microscopy should maintain a supply of malaria RDTs to assist with the initial diagnosis of malaria, and develop standard procedures to ensure prompt confirmation testing by microscopy or PCR. 

In 2007, FDA approved the BinaxNOW malaria rapid diagnostic test for use in the United States by hospital or commercial laboratories (not for use by clinicians or the general public) (*69*). RDTs allow clinical laboratories that do not have malaria microscopy or PCR skills to test patients quickly for malaria instead of sending samples off site for a delayed diagnosis. However, the BinaxNow RDT might not detect malaria in those with very low parasite percentages that would have been detected by microscopy and is limited in its ability to identify all *Plasmodium* species. Microscopy blood smear should be performed for every patient assessed for malaria; good case management requires an assessment of the parasitemia percentage to select the appropriate treatment for either uncomplicated or severe malaria, which is not possible with RDTs or PCR. For surveillance purposes in the United States, a person who has a positive test result by an RDT is considered to have a suspected malaria case, and microscopy, PCR, or both should be performed to confirm the case (*15*). In 2015, five suspected cases that were only determined by RDT were reported to CDC.

CDC provides free diagnostic assistance to laboratories and health professionals diagnosing cases of malaria, including microscopy, PCR testing, and drug resistance marker testing. Increasing the proportion of cases with species confirmation and drug resistance marker testing will improve the epidemiologic understanding of malaria diagnosed in the United States. Public health laboratories should consider developing standard procedures for sending blood samples from persons with malaria to the CDC for molecular surveillance monitoring.

The quality of malaria surveillance data can be limited if data are incomplete or definitions are incorrectly used. CDC determined that 24.2% case reports were missing one or more of the essential variables in 2015, which is significantly higher than in 2014 (21.3%). Of the reports of confirmed cases the United States in 2015, 12.9% were missing the infecting species, 13.1% were missing residence status, and 5.9% were missing the country of acquisition. Incomplete reporting of antimalarial treatments and the infecting species could result in the patient being misclassified as having been treated according to the CDC guidelines. CDC occasionally receives incomplete records for cases only reported electronically through NNDSS; these records contain basic demographic data but not the additional malaria case information from the NMSS case report form. CDC is collaborating with state and local health departments to update the NNDSS reporting platform through the NNDSS Modernization Initiative (NMI). Electronic submission of extended malaria data elements through NMI is anticipated to begin in early 2019. States that implement the malaria NMI surveillance system will no longer be required to submit paper case report forms, which should improve the timeliness and accuracy of malaria surveillance in the United States. State and local health departments are encouraged to report cases using the NMSS case report form until malaria-specific data can be received electronically through NMI. In 2015, a total of 31 (2.0%) cases were not able to be classified, comparable with the number from 2014. All elements on the malaria case report form should be completed as this provides critical information for examining malaria trends that are used to develop recommendations for malaria chemoprophylaxis. Local and state health departments, health care providers, and other health personnel should be vigilant in reporting complete information for malaria cases, especially for essential variables including species, residence, and country of acquisition.

In 2015, the reported number of cases (n = 32) imported from the Dominican Republic increased substantially. In the Caribbean region, endemic transmission of malaria ended in the mid-1960s, except on the island of Hispaniola, which includes the countries of the Dominican Republic and Haiti (*70*). According to NMSS, an average of 3.1 malaria cases were imported per year from the Dominican Republic during 1995–2014 (*71*), with a peak of 11 cases reported in 2007. In 2015, 88% of patients who provided a reason for travel to the Dominican Republic said that they traveled for tourism. Although travel itineraries are not systematically collected, nine persons reported they had traveled to the resort area of Punta Cana, including a cluster of cases acquired during a school trip (*36*,*37*). Only one case imported from the Dominican Republic reported taking chemoprophylaxis to prevent malaria. In response to the significant increase in cases imported from the Dominican Republic, CDC posted a website alert to notify travelers of the numerous malaria cases detected in this area and provided information for prevention, especially chemoprophylaxis (*72*).

In 2015, a total of 11 cases of malaria were imported from Haiti, which is not statistically different from 2014. According to NMSS, an average of 29.1 cases were imported from Haiti into the United States each year during 2000–2009 (*71*). In 2010, the number of imported malaria cases from Haiti reached a peak of 172 cases; this coincided with a magnitude 7.0 earthquake that struck near the capital of Port au Prince and probably reflects the conditions for local transmission as well as an increased volume of travel between the United States and Haiti by relief workers, Haitians, and VFR travelers. During 2010–2014, the number of malaria cases acquired in Haiti has decreased (72 in 2011, 35 in 2012, 23 in 2013, and five in 2014).

Despite the development of chloroquine resistance throughout the world (*73*–*75*), chloroquine remains an effective antimalarial for chemoprophylaxis and treatment of malaria acquired from Hispaniola. Monitoring chloroquine resistance markers is recommended (*76*–*80*), and health care providers should contact CDC to assist with the evaluation of potential chloroquine failures identified after travel to Hispaniola. Seven samples from patients with infections acquired in the Dominican Republic in 2015 were tested for molecular resistance markers, and none contained a genotype associated with chloroquine resistance. Haiti and the Dominican Republic intend to eliminate malaria transmission on the island of Hispaniola by 2020 (*81*,*82*); therefore, the number of cases from both countries is expected to decrease. However, as indicated by the increase in cases in 2015, transmission on the island remains heterogeneous, and malaria cases could begin to increase if efforts to contain transmission are weakened. Prevention, including chemoprophylaxis, is recommended for travelers to Hispaniola, including those going to tourist destinations in the Dominican Republic (*36*,*72*).

NMSS includes information on military personnel who received a malaria diagnosis in the United States. Before 2010, cases among military patients were only reported to CDC by local and state health departments and private health clinicians. However, starting in 2010 CDC collaborated with the Armed Forces Health Surveillance Branch to facilitate direct reporting thus improving opportunities to monitor trends or changes in the deployed military population (e.g., changes in geographical transmission and prophylaxis or treatment failures). From 2014 to 2015, no significant change occurred in the number of military cases reported. Approximately 70.0% of cases diagnosed among military personnel in the United States were acquired in Africa, and more than half (nine cases) of those were from West Africa. The United States responded to the Ebola emergency with the deployment of approximately 3,000 military personnel to Ebola-affected countries starting in September 2014 (*83*). In 2014, two cases in military personnel were diagnosed in the United States with malaria after exposure in an Ebola-affected country (*46*). Despite the increase in troop presence, in 2015, only three cases of malaria were imported to the United States among military personnel after deployment in an Ebola-affected country; one case each was determined to be *P. malariae* and *P. ovale;* one case had an unknown species. All three patients reportedly took atovaquone-proguanil for prophylaxis; the patient with unknown species did not adhere to chemoprophylaxis. These findings are in accordance with other published reports, showing that no U.S. military service members received a diagnosis with *P. falciparum* malaria at international locations after exposure in an Ebola-affected country in 2015 (*64*,*84*,*85*). This achievement is likely because of the resources committed to malaria protection among U.S. military personnel including chemoprophylaxis with atovaquone-proguanil as the preferential antimalarial, insecticide-treated mosquito nets and tents to protect sleeping spaces, skin repellents, and insecticide-treated uniforms (*64*,*86*). In addition, some deployed units were assessed daily for fever and adherence to chemoprophylaxis (*85*). A survey conducted in Liberia during the Ebola response indicated that 96% of service members reported daily adherence to malaria prophylaxis in addition to other prevention measures; this level of chemoprophylaxis adherence is regarded as among the highest reported for deployed U.S. military personnel (*64*). This experience highlights that where malaria risk awareness is high and when chemoprophylaxis and other protection measures are correctly implemented, malaria can be effectively prevented, even among those in high transmission areas and in stressful situations. Although rare, persons who adhere to chemoprophylaxis can become infected with malaria. 

Of the 193 *P. vivax* or *P. ovale* cases in men and in women who were not pregnant at the time of diagnosis (primaquine is contraindicated during pregnancy), 68 (35.2%) received primaquine, the only antimalarial that is active against the dormant parasite liver forms and prevents relapses (*87*). In addition to treatment for acute malaria, all persons who receive a diagnosis of mosquito-acquired *P. vivax* and *P. ovale* and who are not G6PD deficient should receive a course of primaquine for relapse prevention (*20*).

Treatment choices for pregnant women with uncomplicated malaria are limited: mefloquine or quinine plus clindamycin (*20*). However, in April 2017, CDC released a policy note to recommend artemether-lumefantrine for the treatment of uncomplicated malaria among women in the second and third trimester of pregnancy. Artemether-lumefantrine should be considered during the first trimester when other treatment options are unavailable (*88*). This recommendation is consistent with WHO treatment guidelines and reflects current data on treatment efficacy and birth outcomes (*89*,*90*).

During 2000–2014, there were an average of 6.1 deaths from malaria per year, with a low of one death in 2007 and a high of 11 deaths in 2001. In 2015, there were 11 deaths reported from malaria, which was a nonsignificant increase in the number of fatalities compared with 2014, and is similar to the 10 deaths reported in 2013. In 2015, eight of 11 fatal cases were infected with *P. falciparum,* two were infected with *P. vivax,* and one was infected with an unknown species. Severe malaria can best be prevented by appropriate administration of chemoprophylaxis but none of the patients who died from malaria adhered to chemoprophylaxis; one patient reported taking chemoprophylaxis for 8 weeks during 6 months of travel, but stopped it prematurely.

Once malaria has occurred, prompt initiation of treatment is critical—which requires that patients seek evaluation for fever, that care providers consider and test for malaria, and that appropriate treatment is provided rapidly. Four persons did not seek treatment for their illness and were found unresponsive or deceased. Three persons delayed seeking treatment for 3 to 10 days; one patient initially denied travel and malaria was not promptly recognized by her care providers. To facilitate a prompt diagnosis, providers should include malaria in the differential diagnosis of fever in a person who has traveled to a country with malaria transmission, or who is originally from a malaria endemic area. Signs and symptoms of malaria are often nonspecific but typically include fever. Other symptoms include headache, chills, increased sweating, back pain, myalgia, diarrhea, nausea, vomiting, and cough. Health care providers should ask all febrile patients for a travel history. Any delay in the diagnosis and treatment of malaria can result in complications, regardless of the effectiveness of the treatment regimen. Patients suspected of having malaria infection should be evaluated through microscopic examination of thick and thin blood films, as discussed earlier (*91*).

The best approach to prevent severe malaria complications is to provide prompt and appropriate treatment. The choice of an antimalarial treatment regimen should be made on the basis of several factors, including the probable geographic origin of the parasite, the *Plasmodium* species, the percentage parasitemia, and the patient’s clinical status (*68*). Using an oral regimen to treat patients with severe malaria is less effective than parenteral regimens and is not the standard of care. Severely ill patients should be treated aggressively with parenteral antimalarial therapy to ensure rapid adequate drug levels, with the goal to decrease parasitemia to <1% as soon as possible to minimize the likelihood of complications or death (*20*). Quinidine gluconate is the only FDA-approved medication for parenteral malaria therapy but is not available in many hospital formularies. Because parenteral quinidine gluconate is potentially cardiotoxic (e.g., can cause life-threatening arrhythmias and asystole), the drug should be administered in an intensive care setting with continuous electrocardiogram monitoring and frequent blood pressure monitoring. As an alternative to quinidine gluconate, intravenous artesunate is effective to treat severe malaria and it is limitedly available as an investigational new drug through CDC (*45**)*. Artesunate is stocked at nine sites in the United States and can be rapidly shipped at no cost to clinicians; however, the CDC stock of artesunate is limited and interruptions in its availability have occurred (CDC Division of Parasitic Diseases and Malaria, unpublished data, 2017). Certain guidelines and eligibility requirements must be met to enroll a patient in the investigational new drug treatment protocol. Providers who administer artesunate to patients must comply with the investigational new drug protocol and notify CDC of any adverse event after administration (*45*). To enroll a patient with severe malaria in this treatment protocol, health care providers should call the CDC Malaria Hotline at 770-488-7788 or toll-free at 855-856-4713, Monday–Friday, 9 a.m.–5 p.m., Eastern time. After hours, callers should call 770-488-7100 and ask to speak with a CDC Malaria Branch clinician. Travelers and health care providers are encouraged to use CDC resources on malaria prevention and treatment (Table 10) and contact the CDC Malaria Branch for assistance with diagnostic or case management needs.

**TABLE 10:** Sources for malaria prophylaxis, diagnosis, and treatment recommendations

Type of information	Source	Availability	Contact information
Prophylaxis	CDC's Traveler's Health Internet site (includes online access to *Health Information for International Travel*)	24 hours/day	https://wwwnc.cdc.gov/travel
	*Health Information for International Travel* (*The Yellow Book)*	Order from Oxford University Press, Inc. Order Fulfillment 198 Madison Avenue, New York, NY 10016–4314	800-451-7556 or http://global.oup.com/academic
	CDC Malaria Branch website with malaria information and prophylaxis, by country (Red Pages)	24 hours/day	https://www.cdc.gov/malaria/travelers/country_table/a.html
	CDC malaria maps	24 hours/day	https://www.cdc.gov/malaria/map
Diagnosis	CDC Division of Parasitic Diseases and Malaria diagnostic website (DPDx)	24 hours/day	https://www.dpd.cdc.gov/dpdx
	CDC Division of Parasitic Diseases and Malaria diagnostic CD (DPDx)	9:00 a.m.–5:00 p.m. Eastern time, Monday–Friday	dpdx@cdc.gov
Treatment	CDC Malaria Hotline	9:00 a.m.–5:00 p.m. Eastern time, Monday–Friday	770-488-7788 or toll-free 855-856-4713*
	CDC Malaria Clinical Consultation Service	5:00 p.m.–9:00 a.m. Eastern time on weekdays and all day weekends and holidays. For health care providers.	770-488-7100* (This number is for the CDC Emergency Operations Center. Ask staff member to page the person on call for the Malaria Branch.) https://www.cdc.gov/malaria/diagnosis_treatment/treatment.html

* These numbers are intended for health care professionals only.

Patients with severe malaria were significantly more likely to have been inappropriately treated compared with those patients with uncomplicated malaria (40.7% versus 10.0%, respectively), and 37.0% of all pregnant women received treatment contrary to the CDC guidelines for treatment. Health care providers should be familiar with prevention, recognition, and treatment of malaria and are encouraged to consult appropriate sources for malaria prevention and treatment recommendations (Table 10). Health care providers should assess the diagnostic and treatment resources present in their facilities, including availability at night or on weekends. An evaluation of malaria diagnosis capabilities among U.S. laboratories demonstrated that although malaria diagnostic testing services were available to the majority of U.S. laboratories surveyed, very few were complying with all of the current guidelines (*67*). To maintain and improve malaria and other parasitic disease diagnosis capabilities in the United States, CDC conducts training courses several times per year (https://www.cdc.gov/dpdx/index.html). CDC provides several resources for health care providers; care providers seeking assistance with diagnosis (including telediagnosis) or treatment of patients with suspected or confirmed malaria should call CDC’s Malaria Hotline at 770-488-7788 or toll-free 855-856-4713 during regular business hours or CDC’s Emergency Operations Center at 770-488-7100 during evenings, weekends, and holidays (ask to speak to the person on call for the Malaria Branch), or go to CDC’s website at https://www.cdc.gov/malaria/diagnosis_treatment/index.html.

Even though malaria is not endemic in the United States, malaria causes illness and deaths in this country. Imported cases of malaria can reintroduce *Plasmodium* parasites into receptive areas (*11*,*12*,*92*) where the disease is not endemic but potential vectors are present and environmental conditions can support the parasite lifecycle (*10*). The most effective approach for U.S. residents to prevent malaria is to take chemoprophylaxis medication during travel to a country where the disease is endemic. Detailed recommendations for preventing malaria are available to the general public 24 hours per day online at https://wwwnc.cdc.gov/travel/yellowbook/2018/infectious-diseases-related-to-travel/malaria. Malaria prevention recommendations tailored for each country are available online at https://www.cdc.gov/malaria/travelers/country_table/a.html. CDC biannually publishes recommendations in *Health Information for International Travel* (commonly referred to as *The Yellow Book*), which is available and updated on the CDC Travelers’ Health website at https://www.cdc.gov/Features/TravelersHealth.html. (Table 10).

## Footnotes

* The term United States includes all states and territories.

† The number of cases reported for 2014 is different from that published in “Malaria Surveillance — United States, 2014.” This is because of delayed reporting of cases with symptom onset in late 2014 but counted as 2015 cases according to NNDSS definitions. This analysis reclassified all cases according to date of symptom onset.

§ Countries that are considered West Africa include Benin, Burkina Faso, Cape Verde, Côte d'Ivoire, Gambia, Ghana, Guinea, Guinea-Bissau, Liberia, Mali, Mauritania, Niger, Nigeria, Senegal, Sierra Leone, Togo, and West Africa, unspecified.
